# Tanshinone IIA and hepatocellular carcinoma: A potential therapeutic drug

**DOI:** 10.3389/fonc.2023.1071415

**Published:** 2023-01-31

**Authors:** Hu Li, Pengbo Hu, Yajun Zou, Lijuan Yuan, Yucheng Xu, Xiaohui Zhang, Xiaoyan Luo, Zhiqiang Zhang

**Affiliations:** ^1^ Emergency Department, Affiliated Hospital of Binzhou Medical College, Binzhou, China; ^2^ Institute of Medical Science of Binzhou Medical University, Yantai, China; ^3^ Emergency Department, The First Affiliated Hospital of Anhui Medical University, Hefei, China

**Keywords:** Hepatocellular carcinoma, tanshinone IIA, liver fibrosis, nonalcoholic fatty liver disease (NAFLD), MAPK, rapamycin (mTOR)

## Abstract

Because of its high prevalence and poor long-term clinical treatment effect, liver disease is regarded as a major public health problem around the world. Among them, viral hepatitis, fatty liver, cirrhosis, non-alcoholic fatty liver disease (NAFLD), and autoimmune liver disease are common causes and inducements of liver injury, and play an important role in the occurrence and development of hepatocellular carcinoma (HCC). Tanshinone IIA (TsIIA) is a fat soluble polyphenol of Salvia miltiorrhiza that is extracted from Salvia miltiorrhiza. Because of its strong biological activity (anti-inflammatory, antioxidant), it is widely used in Asia to treat cardiovascular and liver diseases. In addition, TsIIA has shown significant anti-HCC activity in previous studies. It not only has significant anti proliferation and pro apoptotic properties. It can also play an anti-cancer role by mediating a variety of signal pathways, including phosphatidylinositol-3-kinase (PI3K)/protein kinase B (Akt)/rapamycin (mTOR), mitogen-activated protein kinase (MAPK), and nuclear factor kappa-B (NF-κB). This review not only reviews the existing evidence and molecular mechanism of TsIIA’s anti-HCC effect but also reviews the liver-protective effect of TsIIA and its impact on liver fibrosis, NAFLD, and other risk factors for liver cancer. In addition, we also conducted network pharmacological analysis on TsIIA and HCC to further screen and explore the possible targets of TsIIA against hepatocellular carcinoma. It is expected to provide a theoretical basis for the development of anti-HCC-related drugs based on TsIIA.

## 1 Introduction

Hepatocellular carcinoma (HCC) accounts for more than 80% of all primary liver cancers. Globally, HCC causes a heavy disease burden and is the main cause of cancer-related deaths in many regions of the world. It is estimated that HCC is the fourth most common cause of cancer-related deaths in the world (1, 2). In China, one person is diagnosed with liver cancer every 67 seconds, and one person dies of liver cancer every 74 seconds (3). Therefore, HCC has become a major public health challenge. The incidence rate of liver cancer varies with gender, etiology, and age and usually occurs in the microenvironment with pro-inflammatory, pro-angiogenic, and pro-fibrotic characteristics. The formation of liver cancer usually develops from chronic hepatitis to cirrhosis, and then from cirrhosis to liver cancer.Liver fibrosis is a repair response to chronic liver injury and is considered a potential pathogenic factor in HCC. Alcoholism, hepatitis C virus infection, and hepatitis B virus infection are the most common causes of liver cirrhosis (3, 4). In fact, the occurrence of liver cancer is not only caused by viral hepatitis but also related to many metabolic diseases. For example, the metabolic syndrome, especially diabetes, also plays a key role in the occurrence and development of HCC. In Europe and the United States, nonalcoholic fatty liver disease (NAFLD) has gradually become a major risk factor for HCC (4, 5).

So far, there are many therapeutic methods for liver cancer, including orthotopic liver transplantation, hepatectomy, ablation technology, interventional therapy, radiotherapy, biotherapy, molecularly targeted therapy, and the use of traditional Chinese medicine. However, these therapies have their own advantages and disadvantages and do not always produce the best patient treatment effect (6–8). For example, although liver transplantation can improve the survival rate of patients to a certain extent, due to the shortage of allografts and lifelong immunosuppression, the clinical promotion of liver transplantation is still greatly limited. In fact, there is still a high recurrence rate and donor HCC incidence rate after liver transplantation. Similarly, hepatectomy also has some shortcomings, such as a low relapse-free survival rate, and patients with advanced cancer and cirrhosis have a high risk of surgery. Although ablation technology is minimally invasive, safe, simple, and relatively cheap, it requires repeated treatment to achieve better efficacy (9, 10), and it is still limited by tumor size and location (8). Although significant progress has been made in the treatment of liver cancer in recent years, most liver cancers were not discovered until they were advanced due to their long incubation period and significant invasive biological behavior. However, biotherapy and molecular targeted therapy are the main treatment methods for the middle and late stages, but the treatment effect is generally poor. At the same time, targeted drugs also have adverse side effects, excessive use may shorten the survival period of patients. In addition, the low sensitivity of HCC cells makes chemotherapy have almost no impact on the overall survival rate of HCC patients (11, 12). Therefore, it is urgent to determine an effective adjuvant method or drug that can effectively treat HCC and further inhibit the progress of HCC.

**Figure 1:**
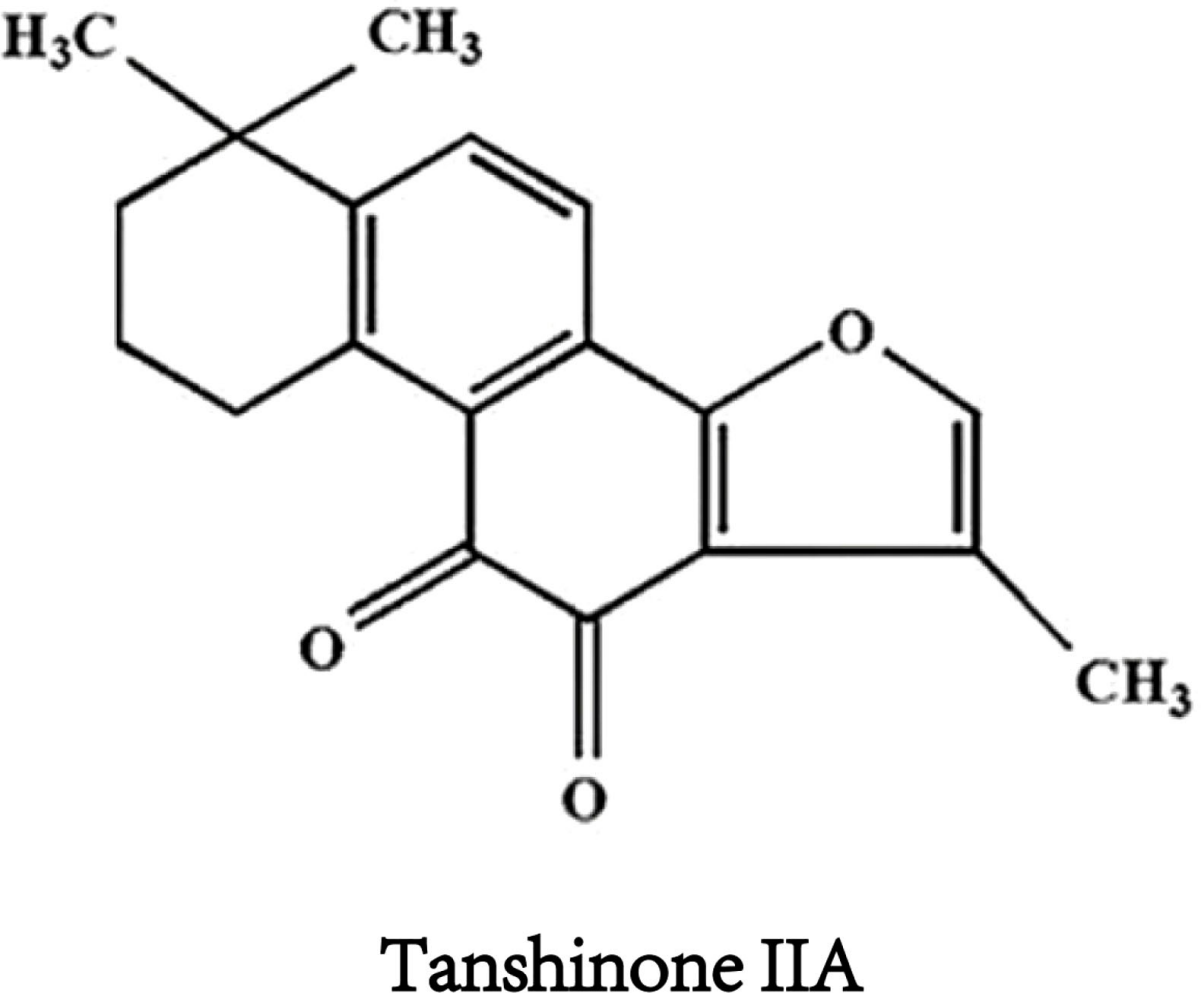
Chemical structure formula of tanshinone IIA.

Tanshinone IIA(**Figure 1**) is the most important effective molecule in fat soluble components of Salvia miltiorrhiza. Since 1934, Japanese scholars first isolated and identified its chemical structure from Salvia miltiorrhiza. TsIIA (C19H18O3, 14,16-epoxy-20-nor-5(10),6,8,13,15-abietapentaene-11,12-dione) Because of its powerful pharmacological effects of anti oxidative stress and anti inflammation (13, 14), it is widely used in China and other Asian countries to treat cardiovascular and cerebrovascular diseases, metabolic syndrome and its complications.The main precursor of TsIIA biosynthesis is geranyl diphosphate (GPP), which originates from the mevalonate and 2-c-methyl-d-erythritol 4-phosphate pathways. GPP is finally transformed into TsIIA and its analogues through a series of downstream enzymes involved in catalytic biosynthesis, including TsIIA, dihydrotanshinone, and cryptotanshinone (15, 16). Interestingly, more and more studies have reported the anti-tumor potential of TsIIA. Previous studies have shown that TsIIA can inhibit the proliferation, metastasis, and progression of various cancer cells (including HCC) by regulating the activation and inhibition of transcription and growth factors, inflammatory cytokines, and intracellular signaling pathways (17–19). However, it is worth noting that more and more studies have reported the effects of TsIIA on HCC cells. We summarize the current research on the effect of TsIIA on HCC *in vitro* in (**Table 1**). These studies reveal their mechanisms of action and their potential as drugs for the treatment of HCC.In fact, TsIIA and its derivatives have long been used in the treatment of liver diseases in Asian countries such as China.

**Table 1:** Tanshinone IIA and hepatocellular carcinoma *in vitro*.

compound	dose	Experimental subjects	IC50	Result/mechanism	reference
TsIIA	40-80μm	HepG,Hep3B	—	TsIIA through up-regulation of miR-30b-p53, PTPN11, and SHP2 signaling pathways induces hepatocellular carcinoma cell death.	(20)
TsIIA	0.125,0.25,0.5,1.0 mg/L	SMMC-7721	—	TsIIA can inhibit the growth and proliferation of SMMC-7721 cells by up-regulating the expression of apoptosis-related genes fas, bax, and p53 and down-regulating the expression of bcl-2 and c-myc.	(21)
TsIIA combined with CYP2A6 inhibitor Met	TsIIA:10μm Met:5μm	Hep1-6	—	TsIIA plays an anti-tumor role by regulating the expression of CYP2A6, thereby increasing the polarization of macrophages	(22)
TsIIA	5,10μg/mL	HepG2	—	TsIIA Induce Nec-1 inhibition and FLIPS regulation mediated apoptosis/necrotic apoptosis.	(23)
1. TsIIA/GA-PEG-SS-PLGA 2. TsIIA/PEG-SS-PLGA 3. TsIIA/PEG-PLGA 4. Free TsIIA	5μm	HepG2	1.TsIIA/GA-PEG-SS-PLGA:1.50 μM 2.TsIIA/PEG-SS-PLGA:2.75 μM 3.TsIIA/PEG-PLGA:2.95μM 4.Free TsIIA:4.96μM	TsIIA can enhance the tumor toxicity and promote apoptosis by up-regulating the level of ROS in cells, increasing the S phase cell cycle block, and up-regulating the expression of caspase 3/7 and P38 proteins.	(24)
TsIIA	0.75mg/mL	MHCC97-H	0.75μg/ml	TsIIA up-regulates or down-regulates 41 proteins in a time-dependent manner (these proteins are critical to cell function and mediate related pathways).	(25)
1. TsIIA 2. Cisplatin 3. Resv 4. l/2TsIIA+1/2Resv 5. l/3TsIIA+2/3Resv	0.5-100 μg/ml	HepG2	Processing 48 hours IC50: 1. TsIIA:40.28+11.63μg/ml 2. Cisplatin:10.14+0.46μg/ml 3. Resv:41.89+11.99μg/ml 4. l/2TsIIA+1/2Resv:10.51+0.24μg/ml 5. l/3TsIIA+2/3Resv:10.39+0.17μg/ml	1. Tanshinone IIA and trans resveratrol synergistically increased the killing effect on human hepatoma cell HepG2, even the killing power was comparable to that of cisplatin. But the side effects are far less than cisplatin. 2. TsIIA plays an anti-cancer role through enhanced apoptosis, sub-G1 cell cycle arrest, and DNA fragmentation.	(26)
1. free TsIIA 2. TsIIA-MSH 3. MSH-TsIIA-PEG	equivalent TsIIA concentration:10 *μ*g/mL	HepG	1. free TsIIA:14.842μg/ml 2. TsIIA-MSH:9.298μg/ml 3.MSH-TsIIA-PEG:6.959μg/ml	TsIIA plays an anti-tumor role through significant inhibition of cell invasion and cell cycle arrest at GO/G1 (DNA replication phase).	(27)
TsIIA	20μM	HepG2,Hep3B	—	TsIIA Significantly reduces the expression of endogenous AKR1B10	(28)
CMT-3 with Tanshinone IIA sodium sulfonate (TSN-SS)	CMT-3:2,5 μmol/l TSN-SS:100 μmol/l	HepG2	—	1. TSN-SS significantly increased the cytotoxicity of CMT-3 in HepG2 cells. 2. TSN-SS significantly reduced the side effects caused by CMT-3.	(29)
Tanshinone IIA mixed micelle(TAN)	50,100,200,400μM	HepG2	—	The significant enhancement on pro-apoptosis by TAN micelles was evidenced by increased chromosome condensation, mitochondria membrane potential loss, cell apoptosis, and cleavages of caspase-3 and PARP.	(30)
TsIIA	0.25,0.5,1.0,1.5,2 mg/L	HepG2,SMMC-7721	—	By reducing the expression of MMP2 and MMP9 and blocking NF- κB activation to inhibit the invasion and metastasis of HCC cells *in vitro*.	(31)
TsIIA	0,1,2.5,5,10,20 μg/m	J5	24h:5.62μg/ml 48h:1.81μg/ml 72h:0.93μg/ml	TsIIA induces J5 cells to stagnate at the G2/M phase and inhibits Hep-J5 cells by increasing the expression of calreticulin, cystatin 12, and GADD153 proteins.	(32)
TsIIA	—	HepG2,Hep3B,PLC/PRF/5,THLE-3	—	It plays an anti-cancer role by regulating the expression of p53 oncogene and inhibiting the overexpression of Pgp.	(33)
TsIIA	—	SMMC7721,MHCC-97L	—	TsIIA inhibits the TGF–β1/Smad pathway, which inhibits cancer cell invasion and metastasis.	(34)
TsIIA	0.01, 0.1, 1, 10, and 100 μmol/l	BEL-7402	24h:1162.0μm 48h:116.1μm 72h:9.7μm	TsIIA induces cancer cell apoptosis by activating calcium dependent apoptosis signal pathway and up regulating metallothionein 1A expression.	(35)
Tanshinone IIA fat emulsion	—	HepG2,SMMC 7721,BEL 7404	—	TsIIA fat emulsion has more stable biological activity, stability and stronger anti-cancer activity.	(36)
TsIIA	0.5 μg/ml	SMMC-7721	—	TsIIA inhibited the proliferation of hepatoma cell line SMMC-7721 and induced apoptosis in a concentration dependent manner, and down regulated the expression of EGF and EGFR.	(37)
TsIIA	0.5,1.0,2.0,5.0,10.0μg/ml	HepG2	24h:14.7μg/mL 48h:7.4μg/mL 72h:3.9μg/mL	TsIIA inhibits cell growth and induces apoptosis in human hepatoma cell line HepG2.	(38)
New PLA Nanoparticles Containing TsIIA (TS-PLA-NPs)	1, 5, 10, 5, 15, 25, and 25 mg/ml	SMMC-7721	24h:16.5mg/ml 48h:7.9mg/ml 72h:6.8mg/ml	TS-PLA-NPs significantly increased the anti-tumor effect of TsIIA.	(39)

IC50:Semi inhibitory concentration, TsIIA/GA-PEG-SS-PLGA: TsIIA-glycyrrhetinic acid coupling poly(ethylene glycol)-disulfide linkage-poly(lactic-co-glycolic acid), MSH-TsIIA-PEG: TsIIA-polyethyleneimine (PEI)-polyethylene glycol (PEG)-coated mesoporous silica nanoparticles (MSN-PEG), TS-PLA-NPs: TsIIA-poly lactic acid-Nanoparticles, SHP2: Src homology region 2-containing protein tyrosine phosphatase 2, Nec-1: Necrostatin-1, CYP2A6: Cytochrome P450 2A6, AKR1B10: Nicotinamide adenine dinucleotide phosphate (NADPH) dependent reductase, CMT-3: Tetracyclin-3.

Similarly, the *in vivo* experiment of TsIIA against liver cancer is also in progress. In the experiment, researchers transplanted human liver cancer cells (HepG2, HCCLM3-RFP and J5, etc.) and mouse H22 liver cancer cells subcutaneously or *in situ* into mice with low immune function to establish animal models. It is finally confirmed that TsIIA can effectively inhibit tumor growth *in vivo*, no matter whether it is injected intravenously, subcutaneously or intraperitoneally. It is worth noting that in the current animal experiments, it has not been observed that TsIIA has toxic effects on the activity of other organs and tissues except tumor tissues. At the same time, we summarized the current *in vivo* experiments of TsIIA and HCC (**Table 2**).

**Table 2:** *In vivo* study of tanshinone IIA and hepatocellular carcinoma.

Animal models	Drug form and dose	Delivery way	Tumor inhibition rate(%)	Result/mechanism	Reference
H22 Mouse Model of Hepatocellular Carcinoma Transplantation	Original TsIIA:1mg/kg TS-PLA-NPs:0.5,1.0,2.0mg/kg	Tail vein injection for 7 days	1mg/kg TsIIA:66.29 0.5,1.0,2.0mg/kg TS-PLA-NPs:70.20,81.08,91.66	TS-PLA-NPs have higher anti-tumor activity than the original TsIIA.	(39)
HepG2 xenograft tumor model in mice	Original TsIIA:15mg/kg ADM:4mg/kg free TsIIA+ADM:15mg/kg+4mg/kg	Subcutaneous injection for 2 weeks (1-7 days, 17-23 days)	TsIIA:32.77 ADM:60.96 TsIIA+ADM:73.18	TsIIA can up regulate the expression of CYP3A4 protein, reduce tumor volume, reduce serum AST level, and thus improve the quality of life.	(40)
HCCLM3-RFP, HepG2-GFP xenograft tumor mouse model	Original TsIIA:1,5,10mg/kg/d	Intraperitoneal injection for 35 days	—	TsIIA can inhibit palliative resection (PR) related enhanced HCC metastasis and inhibit tumor tissue vascular normalization.	(41)
H22 Mouse Model of Hepatocellular Carcinoma Transplantation	TsIIA microemulsion (ME):2,4,8mg/kg	Intravenous injection for 7 days	2mg/kg:0.89 4mg/kg:6.33 8mg/kg:47.66	TsIIA inhibits tumor growth by inducing apoptosis and differentiation of H22 cells.	(42)
J5 xenotransplantation tumor mouse model	Original TsIIA:30 mg/kg 5-fluorouracil(5-FU):30 mg/kg TsIIA+5-FU:30mg/kg+30mg/kg	Intraperitoneal injection for 4 weeks	TsIIA:77.61 5-FU:71.64 TsIIA+5-FU:65.67	TsIIA inhibits tumor growth in J5 xenotransplantation animal models by increasing the expression of Bax and caspase-3 and decreasing the expression of CD31 in mouse models.	(43)
HepG2 xenograft tumor model in mice	TsIIA-mPEG-PLGA-PLL-cRGD nanoparticles(TNPs):1mg/kg	Tail vein injection for 7 days	—	Compared with free TsIIA, TNPs are more cytotoxic to HepG2 cells, and TNPs have a slow release rate of TsIIA. At 120 h, the amount of TsIIA released by TNPs increased to (72.6%) respectively.	(44)
HepG2 xenograft tumor model in mice	Original TsIIA:3,10,30 mg/kg	Subcutaneous injection (5 days a week, 30 days in total)	—	TsIIA exerts its anti-cancer effect directly by inhibiting CYP2J2 activity. The inhibition rate of TsIIA on CYP2J2 is (50%), IC50 is (2.5μM).	(45)
HepG2 xenograft tumor model in mice	1. TsIIA/GA-PEG-SS-PLGA 2. TsIIA/PEG-SS-PLGA 3. TsIIA/PEG-PLGA 4. Original TsIIA The effective concentration of TsIIA is 5mg/kg	Tail vein injection (once every two days for 36 days)	TsIIA/GA-PEG-SS-PLGA>TsIIA/PEG-SS-PLGA>TsIIA/PEG-PLGA>Original TsIIA	The mean tumor volume of free TsIIA, TsIIA-PEG-PLGA and TsIIA-GA-PEG-SS-PLGA groups was 88.7, 66.5 and 38.2% of that of the control group, respectively.	(24)
HepG2 xenograft tumor model in mice	Original TsIIA:1.5g/kg/day,4.5g/kg/day,13.5g/kg/day	Intragastric administration for 5 weeks	Average inhibition rate:48.15 4.5g/kg/day:35.71 13.5g/kg/day:57.14	TsIIA inhibits the growth, invasion and metastasis of human hepatoma cells.	(31)
HepG2 xenograft tumor model in mice	Novel microemulsion of TsIIA:2,4,8mg/kg	intravenous injection for 7days	4mg/kg:34.68 8mg/kg:47.17	A novel drug delivery system, microemulsion enhances the antitumor effects of TsIIA.	(46)

TsIIA/GA-PEG-SS-PLGA : TsIIA-glycyrrhetinic acid coupling poly(ethylene glycol)-disulfide linkage-poly(lactic-co-glycolic acid), MSH-TsIIA-PEG : TsIIA-polyethyleneimine (PEI)-polyethylene glycol (PEG)-coated mesoporous silica nanoparticles (MSN-PEG), TS-PLA-NPs : TsIIA-poly lactic acid-Nanoparticles, CYP3A4: Cytochrome P450 3A4, CYP2J2: Cytochrome P450 2J2.

## 2 Liver protection

The liver is the most important organ for the human body to metabolize and purify toxic substances. However, in the process of exerting its biological function, it may lead to liver dysfunction due to overload, and once dysfunction occurs, liver damage will also occur. In fact, liver injury is often induced by a variety of factors, including chemical pollutants, drugs, alcohol, and microbial infections. Liver injury is considered a highly complex process, accompanied by extensive apoptosis of liver cells. In this process, oxidative stress and inflammatory reactions are generally considered to play a key role (47, 48). In fact, as the main site of heterometabolism, the liver itself is more vulnerable to oxidative damage. Moreover, a large number of studies have confirmed that liver redox status is closely related to the occurrence and development of inflammation, metabolism, and proliferative liver disease. Among them, reactive oxygen species (ROS) play an important role in liver diseases. ROS is mainly produced in the mitochondria and endoplasmic reticulum of liver cells through the cytochrome P450 enzyme (49). Excessive ROS will attack biological macromolecules, such as liver cell proteins, lipids, and DNA, and then lead to abnormal liver structure and function (50). A large number of studies have also confirmed that chronic liver disease is almost always characterized by increased oxidative stress, regardless of the cause. Even oxidative stress plays an important role in the pathophysiological changes that progress to cirrhosis and eventually to hepatocellular carcinoma (HCC) (51, 52). Interestingly, TsIIA has significant antioxidant activity. From the structure of TsIIA, the antioxidant effect of TsIIA mainly depends on the D-ring (furan or dihydrofuran), and the structural change of the D-ring will also affect the antioxidant capacity (53). Secondly, TsIIA can also significantly increase the expression and activity of antioxidant enzymes, such as catalase (CAT), superoxide dismutase (SOD), and glutathione-S-transferase (GST). However, the most important antioxidant stress effect of TsIIA is the activation of the Nrf2 signaling pathway, which has also been confirmed in many studies (54–56).

Although inflammation is a protective reaction of the body, excessive (that is, in terms of intensity and permanent disproportionality) inflammation will lead to the loss of a large number of liver cells, thus aggravating the severity of various liver diseases. These include liver ischemia reperfusion injury, nonalcoholic fatty liver disease (NAFLD), alcoholic hepatitis, liver fibrosis, and even HCC (57). Among them, the overactivation of the classical inflammatory pathway NF-κB plays a key role in liver diseases. NF-κB pathway activation can result in the release of a number of proinflammatory cytokines, including the proinflammatory cytokines TNF-α, IL-1, IL-6, and transforming growth factor-β (TGF-β) associated with liver disease (58). In the liver, TNF-α plays an important role in maintaining the cellular homeostasis of hepatocytes and induces many biological reactions of the liver, such as apoptosis and necrotic apoptosis of hepatocytes, liver inflammation and regeneration, and autoimmunity (59). IL-6 is the most important activator of acute-phase protein expression in hepatocytes. Previous studies have shown that the acute phase protein IL-6 is considered to play an important role in hepatocarcinogenesis because it activates the signal transducer and activator of transcription 3 (STAT3) (60). TGF-β has always been a key factor in the development of liver fibrosis (61). TsIIA can not only directly inhibit the phosphorylation of IB kinase IKK and IKK to upstream molecules, but it can also induce the degradation of IKK and IKK to inhibit NF-κB activation. Moreover, he can also inhibit the activation of the NF-κB pathway by reducing the expression level of other proteins involved in the NF-κB signaling pathway, such as the Toll-like receptor (TLR), myeloid differentiation factor 88 (MyD88), and transferrin 6 (TRF6) (62–64). In fact, in the process of liver injury,because of the unique characteristics of the hepatic sinus structure, the immune response elicited by Kupffer cells was also important in the process of liver injury (65).

### 2.1 Relieve liver ischemia/reperfusion injury

Liver ischemia/reperfusion (I/R) injury is the main manifestation of liver injury after liver transplantation or hemorrhagic shock, with high incidence rate and mortality. The occurrence of I/R involves many molecular mechanisms (66). Interestingly, TsIIA has a significant regulatory role in the mechanism related to liver I/R injury. Previous studies showed that in Qi (67) et al.’s experiment, TsIIA significantly reduced serum transaminase activity in the liver ischemic mouse model pretreated with TsIIA compared with the control group.This liver protective effect is related to TsIIA’s direct inhibition of the level of proinflammatory cytokines in the liver and the reduction of inflammatory infiltration, and these anti-inflammatory effects may be due to TsIIA’s inhibition of TLR4 signaling in liver cells and its regulation of AKT (67). Another study also confirmed this view. In the study of Li (68) et al., they found that compared with the control group, the rat model of orthotopic liver transplantation pretreated with TsIIA (10mg/kg) had significantly lower levels of factors related to the promotion of inflammatory response (TNF-α, IL-4) and significantly higher levels of factors related to the inhibition of inflammatory response (IL-10, TGF-β), and also observed that the apoptosis of hepatocytes was significantly lower than that of the control group.In addition it was also confirmed that this was related to the inhibition of the expression of key molecules of inflammation related signal pathways such as inhibition HMGB1, TLR-4, Myd88, NLRP3 and p-NF-κb p65 released by liver macrophages (KCs).In fact, the protective effect of TsIIA on liver ischemia-reperfusion injury is also attributed to its regulatory effect on liver oxidative stress. In the study of Wang (69) et al., they found that after the preconditioning of TsIIA, the autophagy of MEK/ERK/mTOR pathway in liver cells after liver ischemia-reperfusion was significantly enhanced, and the enhanced autophagy reduced the production of ROS by clearing the damaged mitochondria. This process significantly reduces the infiltration of inflammatory cells, the level of inflammatory cytokines and apoptosis of liver cells, and reduces the damage of liver tissue.

### 2.2 Prevention of toxic chemical liver injury

Liver injury is usually associated with a variety of toxic chemicals, including carbon tetrachloride (CCl4), concanavalin A (Con-A), D-galactosamine (GalN), acetaminophen, and lipopolysaccharide (LPS). The liver injury caused by toxic chemicals is mainly related to an immune reaction and inflammation. They usually lead to large-area apoptosis of liver tissue, which leads to liver fibrosis and finally leads to cirrhosis and HCC (47, 48, 65, 70, 71). Interestingly, a large amount of laboratory evidence shows that TsIIA can significantly improve and promote the repair of liver injuries caused by toxic chemicals. In the *in vivo* and *in vitro* experiments conducted by Park (72), they found that TsIIA can significantly improve the liver injury induced by toxic chemicals (CCl4, GalN, tBH), which is related to TsIIA’s inhibition of processes such as lactate dehydrogenase leakage, GSH depletion, lipid peroxidation, and free radical generation in damaged liver cells. It was also confirmed that the protective effect of TsIIA on the liver was partly attributed to its ability to activate antioxidant related signaling pathways in damaged hepatocytes This has also been confirmed in other studies. In the experiment of Wang (73) et al. TsIIA pretreatment can activate Nrf2 and increase the expression level of Nrf2 target genes, including glutamate cysteine ligase catalytic subunit (GCLC), NAD(P)H, quinine oxidoreductase 1 (NQO1), and blood oxygenase-1 (HO-1), thereby reducing acetaminophen-induced liver injury. Furthermore, the effect of this activity is dose dependent, which is also confirmed in the liver injury model induced by triptolide (74). In fact, the protective effect of TsIIA on the liver is also attributed to the pharmacological effects of immune regulation and the anti-inflammatory reaction of TsIIA. In the experiment of Qin et al., they found that the plasma alanine aminotransferase and aspartate aminotransferase levels in the mouse hepatitis model induced by Con-A pretreated with TsIIA were significantly reduced, and the apoptosis and necrosis of liver tissue were also significantly reduced, and they also found that it was also related to the immune regulation ability of TsIIA.TsIIA increases the ratio of T lymphocyte subsets CD3+, CD4+, and CD8+, significantly reduces inflammatory cytokines, and improves anti-inflammatory cytokine expression (75), which has also been confirmed in other experiments (76, 77). As the most numerous and complex cell type in the body, T lymphocytes are important immune effector cells and regulatory cells (78). Among them, CD3+ cells represent all peripheral mature T cells at the overall level of cellular immunity, and the absolute numbers of CD3+, CD4+, and CD8+ and the ratio of CD4+/CD8+ reflect the cellular immune status. In addition, T lymphocytes can also secrete a variety of cytokines to regulate immune and inflammatory status, such as CD4+ cell subsets. Th cells secrete the cytokines IL-4, IL-5, IL-13, and IL-10, as well as inflammatory factors such as IL-2, IFN-γ, and TNF-α (79). Therefore, the effect of TsIIA on T lymphocyte subsets may regulate the immune state of the body and the release of inflammatory factors, thereby playing an anti-inflammatory role. In addition, they also found that the protective effect of TsIIA on the liver may also be attributed to the inhibition of p38 and NF-κb signal transduction in Kupffer cells by TsIIA because it further alleviates liver inflammation to a certain extent (32) It is worth noting that the water-soluble derivative of TsIIA.It is worth noting that the water-soluble derivative of TsIIA, sodium TsIIA sulfonate (currently the main drug for clinical use of TsIIA), also has the effect of protecting the liver. Like TsIIA, its liver protection is mainly attributed to the regulatory functions of inflammation, oxidative stress and immunity in damaged hepatocytes (80–82).

## 3 Prevent and improve liver fibrosis

The occurrence of liver fibrosis is often mediated by a variety of mechanisms, including hepatitis B and C, alcohol consumption, fatty liver, cholestasis, and autoimmune hepatitis, which can be induced. The liver tissue fibrosis is driven by persistent liver injury and is subsequently characterized by the development of liver cirrhosis (83, 84). It is generally considered to be an excessive wound healing reaction driven by the vicious cycle of hepatocyte necrosis, inflammation, and excessive extracellular matrix (ECM) deposition, and this cycle process has long been recognized as a key step driving the occurrence of HCC (84). Interestingly, previous studies have shown that TsIIA has significant pharmacological activity to improve liver fibrosis. In fact, in China, TsIIA has long been reported to be used in the treatment of liver fibrosis, and its mechanism of action has been studied by scholars at home and abroad (85).

Activation of hepatic stellate cells (HSC) plays a key role in liver fibrosis; activation of HSC plays a key role in myofibroblasts that produce extracellular matrix (ECM) in the liver (83, 86). Interestingly, the antifibrosis ability of TsIIA is partly due to the regulation of HSC. Previous studies have shown that TsIIA can inhibit the proliferation of HSCs in various ways. In the study of Pan (87) et al, they found that TsIIA significantly inhibits the activity of HSCs in rats and leads to HSC apoptosis, which is related to the changes in the expression of proteins related to the regulation of cell apoptosis (PARP, caspase-3, and Bax/Bcl-2 protein ratio increase) and cell cycle (cyclins A, E, and cdk2) in HCC after pretreatment with TsIIA (40). This has also been confirmed in several other studies (88–90). Among them, the C-Raf/probitin complex plays a key role in promoting the activation of the ERK/Bax caspase pathway in this process (87). In fact, TsIIA also involves many molecular pathways to reduce ECM accumulation and HSC proliferation and activation. In two experiments conducted through network pharmacology, it has been preliminarily confirmed that TsIIA treatment can significantly inhibit the expression of c-Jun, pc-Jun, c-Myc, CCND1, MMP9, P65, P-P65, PI3K, and P38 proteins. Through the inhibition of these pathways and proteins, it inhibits the activation and proliferation of HSC, thereby playing a pharmacological role in improving liver fibrosis (90, 91). It is worth noting that this has also been confirmed in another animal study (92), where the antioxidant activity and anti-inflammatory response of TsIIA play a key role (85, 92).

Liver precursor cells, also known as oval cells, are the key to liver tissue repair after liver injury. After severe liver injury, liver oval cells proliferate and differentiate into several lineages, including bile duct epithelial cells, liver cells, etc., which drive the repair of damaged liver cells; therefore, liver precursor cells have significant significance for the treatment of liver fibrosis and cirrhosis (93, 94). Previous studies have shown that the improvement of TsIIA on liver fibrosis is partly attributed to its induction of the proliferation of liver precursor cells. In the experiment carried out by Ze et al., they found that TsIIA can promote Wnt/β-catenin signal transduction significantly related to cell growth, maintenance, and differentiation of stem cells in liver precursor cells, thereby promoting the proliferation of liver precursor cells (95). Similarly, another experiment also confirmed that TsIIA can improve liver fibrosis by promoting the proliferation and differentiation of endogenous stem cells (96).

Cholestasis is characterized by decreased bile flow and bile acid accumulation. It is one of the most common but destructive liver diseases and is significantly related to the occurrence of liver fibrosis (83, 97). Interestingly, previous laboratory data show that TsIIA improves cholestasis significantly. Sodium taurocholate cotransporter polypeptide (NTCP) is one of the main transporters responsible for the reuptake of bile acid (BA). NTCP is mainly expressed on the sinusoid membrane of hepatocytes and transports bound BA from blood to hepatocytes, playing a key role in balancing bile flow (98). However, the pharmacological activity of TsIIA in improving cholestasis is partly due to its regulatory effect on NTCP.In the experiment of Yang (99) et al., they found that TsIIA can significantly promote the transcription and translation of NTCP, thereby promoting the reuptake of BA and to alleviating rifampicin-induced cholestasis. In addition, another mechanism by which TsIIA alleviates cholestasis is to regulate enzymes related to bile acid metabolism (Cyp3a11, Cyp3a13, and Mdr1), and the pathway of this mechanism is mediated by the pregnane X receptor (100). As we all know, schistosomiasis is a special chronic disease that leads to liver fibrosis. Several recent population experiments have shown that the addition of sodium TsIIA IIA sulfonate (STS) to liver protection therapy in the treatment of advanced schistosomiasis fibrosis can help improve patients’ liver fibrosis indicators, avoid adverse reactions, and achieve a good clinical therapeutic effect (101).

## 4 Prevention and improvement of nonalcoholic fatty liver

In recent years, nonalcoholic fatty liver disease (NAFLD) has become the most common chronic liver disease in the world. In some countries, more than 40% of the population is affected. This makes NAFLD a worldwide public health burden and attracts worldwide attention (102). NAFLD is a liver disease driven by multiple factors. Without systematic treatment, nonalcoholic fatty liver disease (NAFLD) usually develops into nonalcoholic steatohepatitis (NASH), leading to large-area apoptosis and necrosis of liver cells so that liver fibrosis occurs, leading to cirrhosis and HCC (102–104). However, a large number of previous laboratory data show that TsIIA is a potential compound for NAFLD prevention and treatment, and its mechanism of action may be related to the regulation of intracellular molecular targets (such as PPAR, CYP1A2, and MMP2), thus regulating lipid metabolism, antioxidant activity, and anti-fibrosis (105). TsIIA has been shown in the literature to reduce apoptosis in a fatty liver model by improving liver antioxidant capacity and improving liver steatosis (105–107). In addition, the pharmacological effect of TsIIA on inhibiting inflammation also plays a key role. Abnormality and excessive accumulation of lipid droplets in liver cells are the main characteristics of steatosis and NAFLD, or metabolism-related fatty liver disease (MAFLD). The disorder of lipogenesis contributes to liver steatosis and plays an important role in the pathological progress of MAFLD (103). Interestingly, a large body of laboratory evidence suggests that TsIIA can improve non-alcoholic fatty liver by regulating the expression and activity of lipid metabolism enzymes in liver cells. In the experiment of Jia et al., they found that TsIIA can regulate lipid metabolism by regulating the expression of the transforming enzyme (subtilisin/kexin 9 type) signal pathway protein of miR-33a/SREBP-2 (sterol regulatory element binding protein 2)/Pcsk9 pre protein, thus improving fatty liver. This may be because TsIIA reduces the expression of the liver X receptor α (LXRα)-mediated lipogenesis gene and lipid droplet accumulation (63). Interestingly, STS has also been reported to have the same therapeutic effect (108, 109). In addition, TsIIA has also been reported to inhibit the overactivation of endoplasmic reticulum stress pathway-related protein molecules (phosphorylation of eIF2α, expression of GRP78, ATF6, and CHOP), so as to improve palmitate-induced hepatocyte degeneration (110). It is worth mentioning that TsIIA has also been reported to have a regulatory effect on alcohol-induced fatty liver. TsIIA can effectively prevent ethanol-induced fat accumulation, reduce LPS and ethanol-induced Kupffer cell sensitization, inhibit the synthesis of reactive oxygen species and nitrogen, inhibit fatty acid synthesis, and stimulate fatty acid oxidation, thereby improving fatty liver (111).

NAFLD is usually driven by many factors, among which metabolic syndrome is the most common. Metabolic syndrome not only significantly promotes the occurrence and progression of NAFLD but also plays a key role in the occurrence and progression of HCC (103, 112). In fact, many studies believe that NAFLD is the liver manifestation of metabolic syndrome (113). Because primary hepatic steatosis in NAFLD is associated with many risk factors of the metabolic syndrome, such as obesity, insulin resistance, dyslipidemia, hypertension, and type 2 diabetes. And these risk factors are the key features of the pathophysiology of the metabolic syndrome and NAFLD (114). In addition, the liver is the place where fasting blood glucose, very low-density lipoprotein, and most triglycerides (TG) are produced. The metabolism of these products is also a key component of the metabolic syndrome. TG and free fatty acids (FFA) in particular can cause excessive accumulation of liver fat and inflammatory liver injury (115). Moreover, some studies have found that metabolic syndrome and NAFLD may have the same risk distribution. For example, women with a waist circumference greater than 88 cm and men with a waist circumference greater than 102 cm have an increased risk of NAFLD by 4.9 times, fasting blood glucose by 2.1 times, hypertension by 1.8 times, and hypertriglyceridemia by 1.6 times (116). During the development of NAFLD, it is generally believed that, hepatic insulin resistance will lead to the occurrence and development of NAFLD, which seems to be partly because the liver cells lose the ability to respond to insulin, which makes them unable to effectively inhibit the production of glucose but retain the ability to drive fat production and increase new fat production, leading to abnormal accumulation of liver fat (117). Therefore, many compounds can inhibit the occurrence of NAFLD by improving the insulin resistance status of the body.In fact, TsIIA also has a powerful pharmacological effect on improving insulin resistance. The existing literature shows that TsIIA can reduce the blood glucose level and body weight of experimental mice without changing food intake, which is attributed to the inhibition of phosphorylation of PERK and JNK by TsIIA and the enhancement of Akt phosphorylation mediated by insulin, as well as the uptake of glucose through AMPK activation under ER stress (118, 119). Interestingly, the NF-κB-induced AMPK signaling pathway seems to be involved in this process (120).

## 5 Effect on hepatocellular carcinoma

### 5.1 Prevention of toxic chemical liver injury

#### 5.1.1 Induction of apoptosis in hepatocellular carcinoma cells

Apoptosis refers to the process of spontaneous cell death regulated by genes, which plays an important role in maintaining the homeostasis of the internal environment and the normal growth and development of organisms.It is reported that the occurrence and progression of tumors often indicate an imbalance between apoptosis and proliferation. However, the signal molecules and proteins related to apoptosis have been regarded as effective targets for cancer prevention and treatment by scientists and have been extensively studied by the scientific community for a long time (121, 122). Interestingly, previous studies have shown that TsIIA can induce apoptosis of HCC cells by changing the balance between the expression of pro-apoptotic proteins and anti-apoptotic proteins in the B cell lymphoma 2 (BCL-2) family of HSCs (35, 38). Secondly, caspase, which contains cysteine, is the key enzyme to execute cell apoptosis. Among them, caspase-3 is the key executor of mammalian cell apoptosis (122). TsIIA can significantly induce not only its expression and phosphorylation in HCC, but also the activation and expression of its upstream promoter Caspase-8 or Caspase-9. However, the regulatory effect of TsIIA on the above proteins is partly through the miR30b-p53-PTPN11/SHP2 signal pathway (80). In fact, the inhibition of cytochrome P450 in HCC cells by TsIIA also plays a key role.TsIIA not only leads to apoptosis of HCC by inhibiting cytochrome P450, but macrophage polarization can be induced by inhibition of cytochrome P450, thereby promoting the killing effect of macrophages on HCC (22, 45). In addition, other mechanisms are involved in the apoptosis of HCC cells induced by TsIIA. It is reported that TsIIA also has a significant regulatory effect on apoptosis-related genes in HCC cells. The expression of apoptosis-related genes bcl-2 and c-myc in HCC cells pretreated with TsIIA is down-regulated, and the expression of fas, bax, and p53 is up-regulated. Changes in these genes can often promote the apoptosis of cancer cells (21). Secondly, TsIIA has also been proven to up-regulate the expression of key proteins in endoplasmic reticulum stress-induced apoptosis, including glucose regulatory protein 78 (BiP/GRP78), endoplasmic reticulum (ER) stress sensor (IRE1-α), and its downstream target CAAT/enhancer binding protein homologous egg white/growth arrest, and DNA damage inducing gene 153 (GADD153/CHOP), It leads to apoptosis of HCC cells through ER stress (32). Interestingly, TsIIA has also been reported to have a significant regulatory effect on calcium-dependent apoptotic signals. In the experiment of Dai et al., they found that the level of calcium ions in HCC cells treated with TsIIA was significantly up-regulated and the gene expression and mitochondrial membrane potential of metallothionein 1A (MT1A) were significantly reduced. The above data showed that TsIIA induced HCC apoptosis in part through the calcium-dependent apoptosis pathway (35). It is worth noting that Nec-1 inhibition and FLIPS [FLICE (Fas related death domain like interleukin-1β converting enzyme)] - inhibition of protein mediated apoptosis/necrotic apoptosis also play a key role in the process of apoptosis induced by TsIIA in HCC (77). In addition, the proliferation effect of cancer cells, mediated by epidermal growth factor (EGF), is also inhibited by TsIIA. TsIIA not only inhibits the expression of EGF and its receptor (37), but also inhibits the expression of AKR1B10 and the upregulation of promoter activity induced by it, which is related to the inhibition of transcription complex AP-1 by TsIIA (28).

#### 5.1.2 Induction of hepatocellular carcinoma cell cycle arrest

A normal cell cycle is essential for the homeostasis of the internal environment and the normal development of organisms. The disorder of this system often leads to uncontrolled cell proliferation, which leads to the occurrence of tumors.The cell cycle’s normal progression is dependent on the regulation of various cyclins, cell cycle dependent protein kinases, and cell cycle inhibitors. In mammals, cell cycle processes are orchestrated by molecular mechanisms centered on the phase-specific expression of cyclins. They form complexes with specific cyclin-dependent kinases (CDK) and promote the phosphorylation of the retinoblastoma tumor suppressor (Rb) to allow the cell cycle to progress to the next stage (123–125). Therefore, in order to maintain their biological characteristics (uncontrolled proliferation), tumor cells often induce the expression of proteins that drive cell cycle progress and the loss of genes encoding cell cycle inhibitors or inactivate cell cycle regulators such as Rb and P53 (125, 126). Interestingly, the current literature shows that TsIIA can significantly inhibit the proliferation of HCC by inducing HCC cell cycle arrest. Its mechanism is that it can significantly down-regulate cyclin D1, A, and E in HCC cells (80, 82, 88, 89). This seems to be partly due to the fact that TsIIA can directly inhibit the phosphorylation of STAT3 Tyr705, a proliferation-promoting signal pathway in cancer cells (127). In addition, TsIIA also has a significant ability to induce CDK-like proteins in HCC cells. Previous studies have shown that TsIIA can not only inhibit the expression of CDK in HCC cells but also increase the activation and expression of CDK inhibition (p21, p27, p16) by promoting p53 and miR30b-p53 PTPN11/SHP2-mediated methods (80). In fact, TsIIA also showed an inhibitory effect on the hyperphosphorylation of Rb (91). These mechanisms promote the G1 phase arrest of cancer cells, and this seems to be partly attributed to the antioxidant effect of TsIIA (24). In addition, the normal progress of the cell cycle also depends on the regulation of other cell factors. These regulatory factors are not only important for the maintenance of genome stability and integrity but also participate in the formation and regulation of structures such as spindles that maintain the normal progress of mitosis. Interestingly, the existing literature shows that TsIIA and its derivatives also show regulatory effects on these factors in HCC cells. This includes the expression of the induced arrest and DNA damage-inducing protein (GADD45A), the reduction of the activity of Polo-like kinase 1 (PLK1), and the expression of checkpoint-related proteins. Finally, the influence of TsIIA on these proteins and factors eventually leads to the arrest of the HCC cell cycle (128).

### 5.2 Inhibition of metastasis and invasion of hepatocellular carcinoma cells

**Figure 2:**
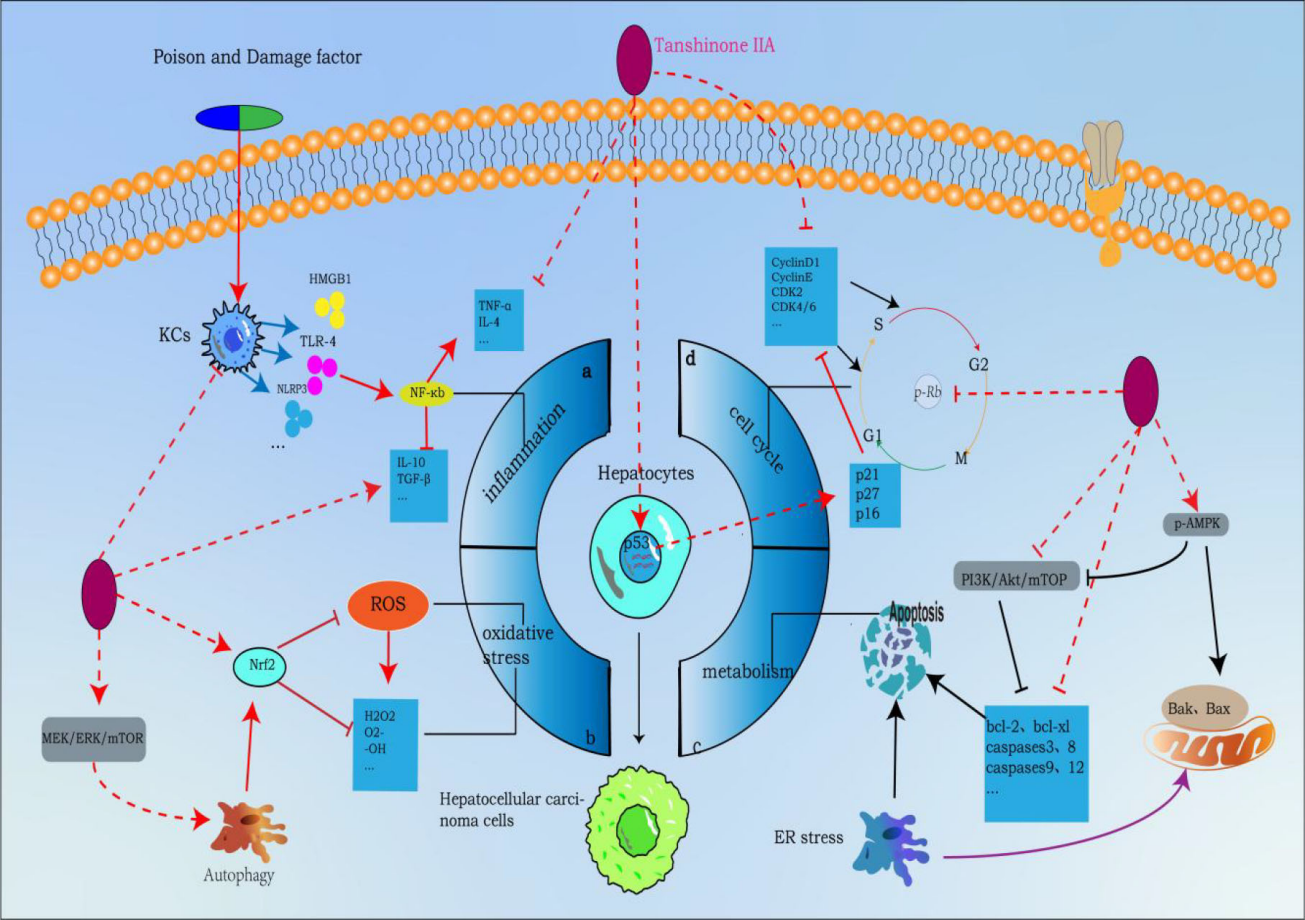
Tanshinone IIA inhibits the molecular process of hepatocellular carcinoma (induced by Tanshinone IIA are noted by using →, while the inhibition represented by ⊣ symbol). The occurrence of hepatocellular carcinoma is closely related to **(A)** inflammation, **(B)** oxidative stress, **(C)** cell cycle and **(D)** metabolic apoptosis.1. Tanshinone IIA can block a by inhibiting NF-κB upstream activators released by liver macrophages (KCs), inhibiting NF-κB signaling pathway, inhibiting proinflammatory factors and promoting the release of proinflammatory factors to block process a;2. Tanshinone IIA can mediate autophagy to activate Nrf2 through MEK/ERK/mTOR pathway to eliminate ROS and other oxygen free radicals to block process b;3. Tanshinone IIA blocks process c by regulating apoptotic proteins through PI3K-AKT-mTOR and p-AMPK pathways;4. Tanshinone IIA blocks process d by regulating cyclin p-Rb and p53 genes.

Metastasis of tumor cells is often one of the main causes of death. Previous studies have shown that HCC can metastasize through a variety of mechanisms. Cancer cells can metastasize by secreting matrix metalloproteins (MMPs), dissolving extracellular matrix, promoting angiogenesis, recruiting certain cytokines and chemokines, and activating relevant molecular signals (129, 130), which brings great challenges to clinicians’ diagnosis and treatment. TsIIA has been shown in the literature to be an effective inhibitor of HCC metastasis and invasion in order to prevent HCC metastasis. EMT refers to a variety of changes in cells at the molecular level. It is a process in which epithelial cells lose their top base polarity and cell-cell adhesion and migrate to invasive mesenchymal cells. The cells receiving EMT showed that the expression level of epithelial genes (such as E-cadherin, ZO-1, and occludin) was decreased while the expression level of mesenchymal genes (such as N-cadherin, vimentin, and fibronectin) was increased. In most cases, the loss of E-cadherin was a sign of EMT. Changes in gene expression during EMT lead to many phenotypic changes, such as changes in cell morphology, loss of adhesion, and acquisition of stem cell-like features (95, 96). It is known that several key signaling pathways including transforming growth factor β (TGFβ), Wnt, Notch and Hedgehog are involved in EMT (131, 132). It is interesting that TsIIA in HCC cancer cells shows the function of regulating EMT. Previous laboratory data showed that the expression level of E-cadherin in HCC cells treated with TsIIA was increased, while the expression levels of N-cadherin and vimentin were decreased. However, the main reason for the above mechanism is that the proportion of Smad7/Smad3 is up-regulated, which inhibits the TGFβ-mediated MET process (34, 133). This was confirmed in the *in vitro* and *in vivo* models constructed by Ma et al. with hepatoma cells Bel-7404, SMMC-7721, and Bel-7402. TsIIA promotes the apoptosis of hepatoma cells and inhibits cell proliferation, invasion, and migration by up-regulating the protein expression of SMAD7 mRNA *in vitro* and *in vivo* in a time- and dose-dependent manner. Moreover, it also reduced Ki67 (a marker reflecting tumor proliferation and high invasiveness) (133). In fact, TsIIA also showed a significant regulatory effect on other MET-related signal pathways, such as Wnt, STATE3, etc. At the same time, TsIIA can also inhibit the metastasis of HCC cells by inhibiting selectin-induced EMT (55, 134). In addition, matrix metalloproteinases have long been proven to be significantly related to cancer cell metastasis. This factor can promote cancer cell metastasis by dissolving the cancer cell matrix (129), while TsIIA has long been proven to inhibit the expression of MMP-3/9 in HCC, which seems to be partly due to the blocking of the activation of NF-kappa B (31). This was verified in the study of Xu et al., when the human hepatoma cells HepG2 and SMMC-7721 were treated with 0, 0.25, 0.5, 1, 1.5, 2mg/L TsIIA for 96 hours. The number of invasive HepG2 cells was 62 ± 5,58 ± 8,39 ± 6,30 ± 5,26 ± 7,21 ± 5, and the number of invasive SMMC-7721 cells was 79 ± 8,75 ± 13,50 ± 12,40 ± 7,31 ± 7,25 ± 4. In addition, *in vivo* and *in vitro* experiments on inhibition of tumor metastasis. They found that when HepG cells were treated with 2mg/L for 96 hours, the number of HepG2 cells migrated was only 37 ± 5, while the blank control group was 115 ± 7. Second, in their *in vivo* experiments. When mice were treated with different doses of TsIIA (0 g/kg/day, 1.5 g/kg/day, 4.5 g/kg/day, 13.5 g/kg/day), the number of tumors visible on the lung surface of mice was observed: 14 ± 5, 12 ± 4, 9 ± 4, 6 ± 3 (31). In addition, it is worth noting that TsIIA also shows a significant therapeutic effect on the recurrence of liver cancer. In the mouse model after palliative resection of HCC, TsIIA inhibits the recurrence and metastasis of residual cancer by inducing vascular normalization (by inducing endothelial cell normalization and regulating the expression of angiogenic factors) (41) (**Figure 2**).

### 5.3 As a synergist of anticancer drugs

TsIIA seems to have great potential as a synergist of anti-cancer drugs. Previous literature shows that the combination of several anti-cancer drugs (adriamycin (ADM), sorafenib and its derivatives SC-1, trans resveratrol (RESv), 5-fluorouracil (5-FU), and TsIIA significantly enhanced the anti-tumor effect of anti-cancer drugs (26, 33, 40, 135, 136). In fact, previous studies have confirmed that TsIIA is used in combination with cisplatin to treat prostate cancer (137), with imatinib to treat myeloid leukemia, and with 5FU to treat colon cancer (138, 139). Among them, in the research on TsIIA and HCC, Liu et al. conducted an *in vivo* experiment with a tumor-bearing mouse model constructed by human hepatocellular carcinoma HepG2 cells. They found that when the original free form of 15 mg/kg TsIIA was combined with 4 mg/kg ADM, compared with the mice treated with 15 mg/kg TsIIA and ADM alone, the combined treatment had a significant anti-tumor effect of 73.18%, while the use of 15 mg/kg TsIIA and ADM alone was 60.96% and 32.77%. In addition, the tumor weight gain rate of TsIIA combined with ADM was the lowest, only 17.30% (40). In addition, they also found that this is related to the regulatory effect of TsIIA on cytochrome P450 (CYP) and cytochrome P450 3A4 (CYP3A4) (40). As we all know, CYP, as a superfamily of enzymes with many isoenzymes, is closely related to drug metabolism and plays an important role in drug detoxification, cell metabolism, and body balance (140). Among them, the CYP3A subfamily is the most abundant among all CYP subtypes and can catalyze the oxidative metabolism of various clinical drugs. CYP3A4 is the most important member of CYP3A and one of the most important subgroups of CYP (141). It has been found in previous studies that the gene expression of CYP3A4 will decrease when HCC has venous invasion, intrahepatic metastasis, and early recurrence. Therefore, downregulation of the CYP3A4 gene is considered an independent predictor of survival and early recurrence in HCC patients (142). However, interestingly, TsIIA combined with ADM increased the concentration and activity of the cytochrome P450 enzyme and up-regulated the expression of the CYP3A4 protein. In addition, TsIIA also plays a key role in promoting the normalization of tumor vascular system structure and function. In the study of Zhang et al., they found that after pretreatment with TsIIA, the vascular wall structure of tumor tissue was better, the pericellular coverage increased, and the contact between basement membrane and endothelial cells increased, which reduced the outflow of polyethylene glycol liposome doxorubicin (PLD), increased the distribution concentration of PLD, and promoted its anti-tumor effect (136). In addition, Lee et al. found in the comparative experiment of four tanshinones (cryptotanshinone, dihydrotanshinone, tanshinone I, tanshinone IIA) in combination with doxorubicin to play an anti HCC role. When four tanshinones (0-25µM) were used in combination with doxorubicin, HepG2 cells were strongly inhibited and cytotoxic. Among them, four tanshinones can cause caspase-3 activation at 25µM and induce apoptosis of cancer cells. Moreover, TsIIA has a wide concentration range (1.56 to 25µM) in doxorubicin induced growth inhibition of HepG2 cells, and shows the largest synergistic effect at 25µM. However, this is due to TsIIA enhancing the retention of doxorubicin in HCC cells with overexpression of the cell multidrug resistance (MDR) related gene Pgp (33). In fact, MDR is one of the main mechanisms by which tumors obtain drug resistance, and it is the reason for the failure of many chemotherapy and targeted drugs (143). However, it is interesting that the existing literature shows that TsIIA can enhance the anti-tumor effect of tumor drugs by downregulating the expression of MDR-related genes (P glycoprotein, topoisomerase, lung cancer resistance protein). This was confirmed in the experiments of Li et al. using TsIIA combined with doxorubicin to treat breast cancer, and Su et al. using TsIIA combined with 5-FU to treat Colo205 colon cancer cells (139, 144).

Of course, the combination of TsIIA and anticancer drugs can also significantly inhibit the migration and invasion of HCC. In the research of Qiu et al., 1.5 µg/mLTsIIA combined with 5 µM sorafenib and 1.5 µg/mLTsIIA combined with 5µM sorafenib derivative SC-1 were compared with sorafenib alone or its derivative SC-1. TsIIA in combination with sorafenib reduced Huh7 (21.3% ± 1.3%), and HepG2 (116% ± 13.7%) cell migration when compared to the control group. TsIIA combined with SC-1 also reduced Huh7 (6.1% ± 0.7%) and HepG2 (24% ± 4.6%) cell migration. In terms of inhibiting cell invasion, TsIIA combined with sorafenib reduced Huh7 (27.1% ± 2.5%) and HepG2 (19.7% ± 2.5%) cell invasion compared with the control group. TsIIA combined with SC-1 reduced Huh7 (34.3% ± 4.4%) and HepG2 (12.9% ± 4.4%) cell invasion (135). Secondly, they also found that the combination therapy inhibited the growth, metastasis, and invasion of HCC cells by inhibiting the STAT3 signal. STAT3 is a protein transcription factor composed of 770 amino acids that can regulate many genes related to apoptosis and epithelial mesenchymal transformation (EMT) and plays an important role in tumorigenesis, immune regulation, and the tumor microenvironment (145). It has been found in previous studies that STAT3 is constitutively activated in many tumors, including HCC, and may increase after long-term treatment with sorafenib in HCC cells. Therefore, effective inhibition of the STAT3 signal is essential for HCC treatment (146). It is worth noting that TsIIA combined with trans resveratrol (RESv) can produce an anti-tumor effect comparable to cisplatin. In the study of Zhang et al., when HepG2 cancer cells were treated with a certain concentration (2µg/ml increased to 40µg/ml) of TsIIA, RESv, cisplatin, l/2TsIIA+1/2Resv, l/3TsIIA+2/3Resv for 24 hours. The IC50 of each component after 24 hours was: TsIIA group: >100µg/ml, RESv group: 74.59 ± 1.62µg/ml, cisplatin group: 35.92 ± 2.14µg/ml, l/2TsIIA+1/2Resv group: 53.61 ± 5.34µg/ml, l/3TsIIA+2/3Resv group: 37.07 ± 1.01µg/ml. Interestingly, this data shows that the IC50 value of l/3TsIIA+2/3Resv is close to that of cisplatin alone. Moreover, the combined treatment of TsIIA and Resv can enhance the effects of cancer cell apoptosis, subg1 cell cycle arrest, and DNA fragmentation. The most important thing is that this combination therapy has no side effects, and it can avoid hemolytic anemia, nephrotoxicity, neurotoxicity, ototoxicity, bone marrow toxicity, and other serious side effects caused by the use of cisplatin alone (26).

## 6 Network pharmacological analysis

**Figure 3:**
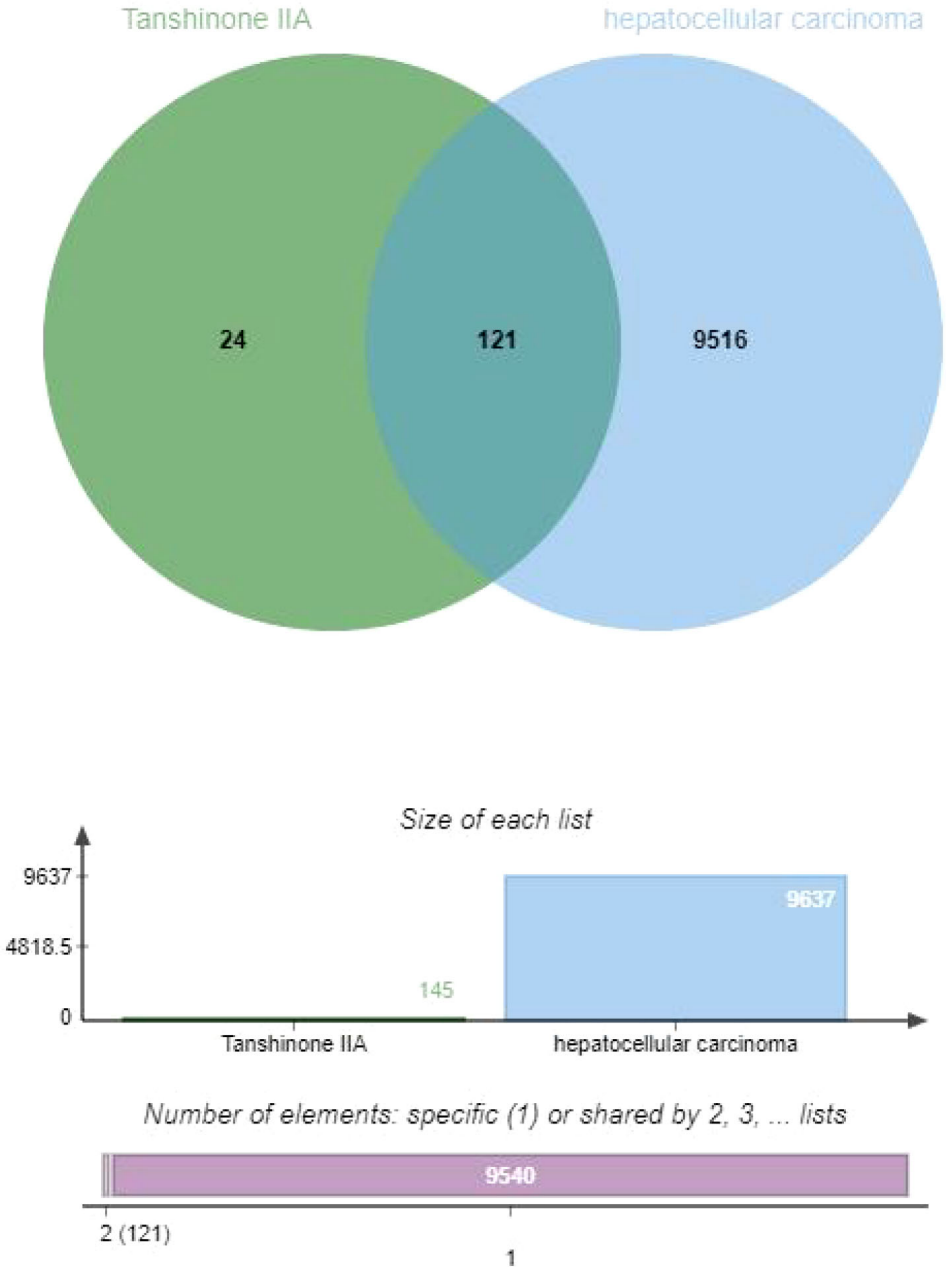
Venny diagram of tanshinone IIA and hepatocellular carcinoma.

In order to explore and verify the action target and molecular pathway mechanism of TsIIA on HCC, we specially studied the network pharmacological analysis of TsIIA and HCC.First, after determining the structural formulas of Tanshinone IIA through Pubchem(https://pubchem.ncbi.nlm.nih.gov), we screened drug targets using the Swiss target prediction database(http://www.swisstargetprediction.ch/) and the traditional Chinese Medicine System Pharmacology database(https://www.tcmsp-e.com/), and submitted the collected targets to the UniProt database (https://www.uniprot.org/), limiting the species to “Homo sapiens”, converting the protein targets into official gene names, select gene targets with probability greater than 0 in the Swiss target prediction database, and obtain drug target genes: Tanshinone IIA (145), after excluding duplicate genes. Secondly, we searched the Genecards(https://www.genecards.org/) and Disgenet databases(https://www.disgenet.org/) by using the keyword “Hepatocellular carcinoma” to obtain disease targets, and obtained 9637 disease target genes after excluding duplicate targets in the two databases. Then, we input the drug target genes and disease target genes obtained by the above methods into the online Venny 2.1 mapping platform(https://www.bioinformatics.com.cn/)to obtain the cross target genes of “Hepatocellular carcinoma” and “Tanshinone IIA” 121(**Figure 3**).

**Figure 4:**
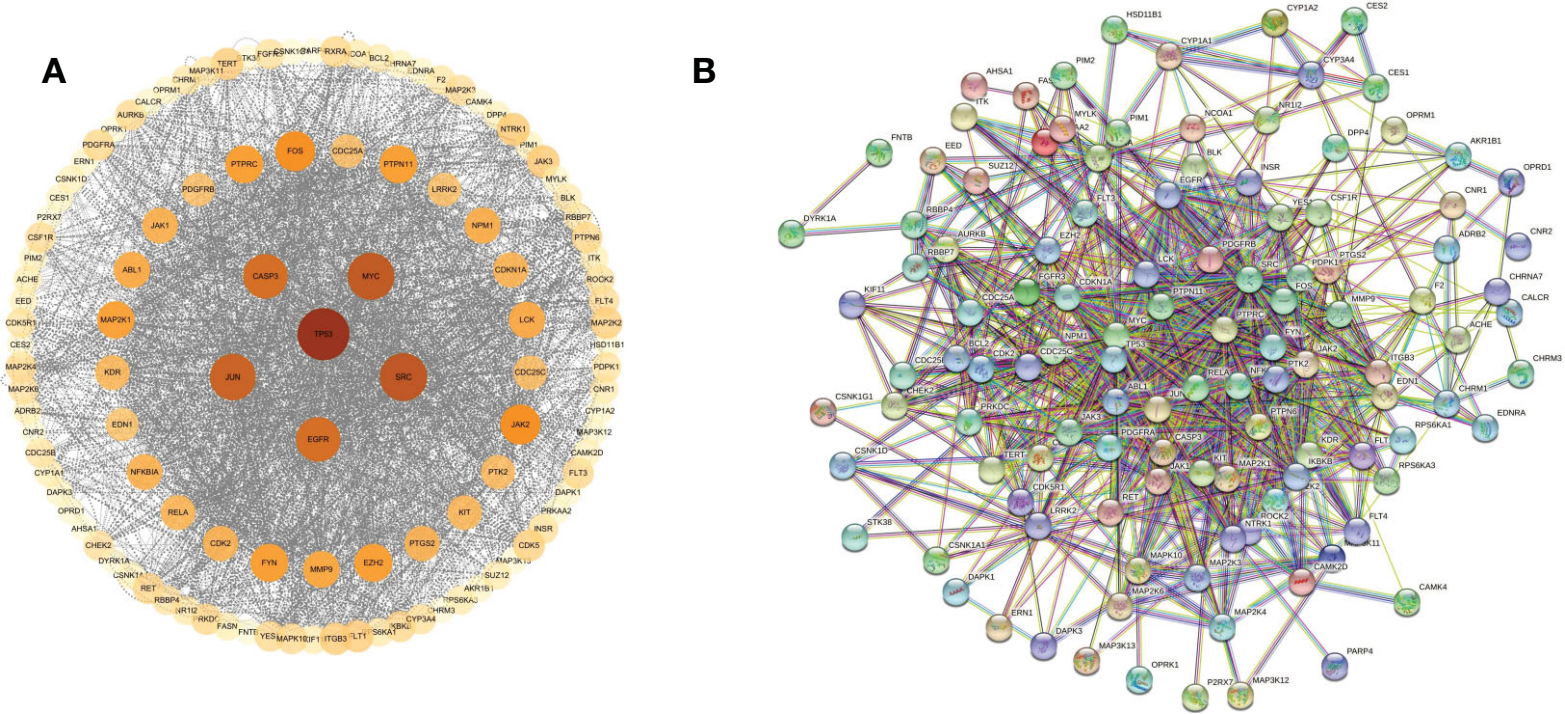
Protein network. **(A)** Tanshinone IIA and hepatocellular carcinoma protein network analysis, **(B)** Tanshinone IIA and hepatocellular carcinoma protein interaction network.

**Figure 5:**
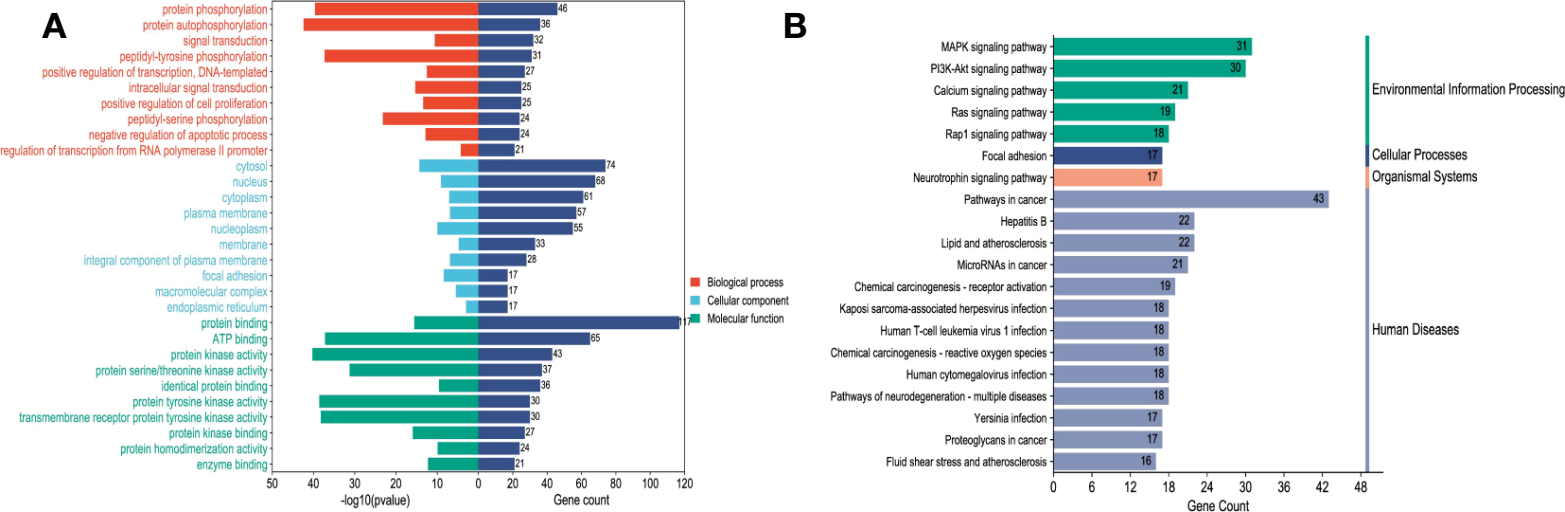
Enrichment analysis.**(A)** Tanshinone IIA and hepatocellular carcinoma GO enrichment analysis, **(B)** Tanshinone IIA and hepatocellular carcinoma KEGG enrichment analysis.

These cross genes are considered as possible targets for the treatment of HCC by TsIIA, and we have analyzed them through a series of methods.First, we uploaded these genes to the String online database (https://string-db.org/) to form a protein-protein interaction map. We set the species as “human”and the comprehensive score > 0.4 is the critical value for inclusion in the network. Then we further visualized these results with the help of Cytoscape 3.9.1 (**Figure 4**) to find the key targets of TsIIA.At the same time, we also analyzed the function of gene ontology (GO) and the path of Kyoto Encyclopedia of Genes and Genomes (KEGG). After inputting these gene data into the David data platform(https://david.ncifcrf.gov/tools.jsp) and setting the species as “Homo species”,we further analyzed the enrichment analysis of TsIIA on HCC related biological processes (BP), cell components (CC), molecular functions (MF) and signal pathways. For the obtained information, we meet the p-value<0.05, and select the first 10 enrichment information of BP, CC and MF and the first 20 enrichment information of KEGG according to the sequence of gene number, and use bioinformatics online platform(https://www.bioinformatics.com.cn/)Visualize the analysis results (**Figure 5**).

**Figure 6:**
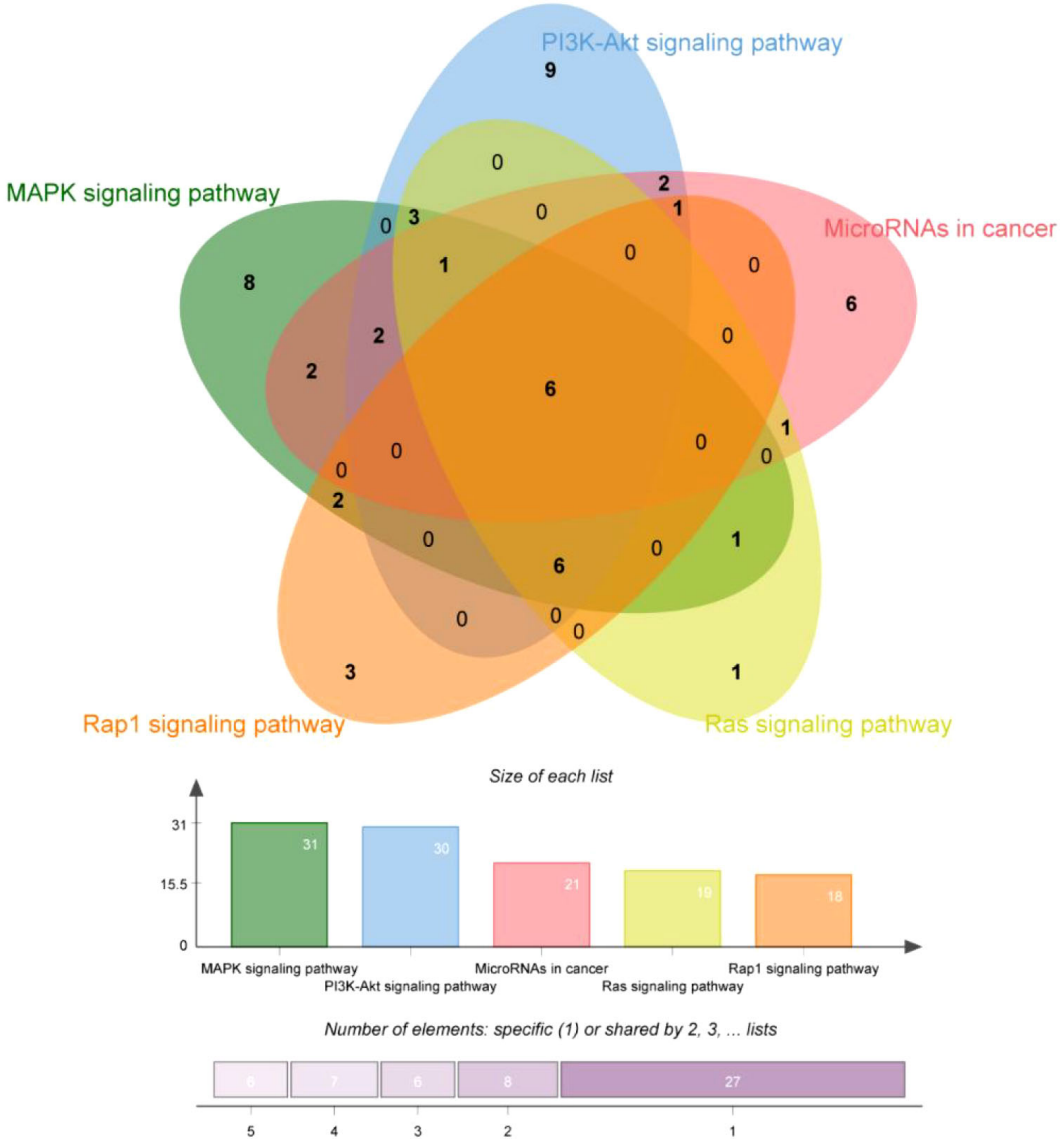
Key pathway enriched genes. Gene interaction map of TsIIA on PI3K/Akt signaling pathway, MAPK signaling pathway, Ras signaling pathway, rap signaling pathway and microRNA regulatory genes in HCC.

Finally, our results show that TsIIA has a very high targeting activity for HCC. Among them, the cell tumor antigen p53 (TP53), myc proto-oncogene protein (myc), transcription factor AP-1 (JUN), Proto-oncogene tyrosine-protein kinase SRC(SRC), caspase-3 (CASP3), and epidermal growth factor receptor(EGFR) play a key role in the anti-HCC process of TsIIA. In addition, previous studies have confirmed that TsIIA, as a fat-soluble polyphenol, can enter cells directly through the cell membrane due to its fat-soluble property and rely on the direct combination of the D ring in the molecular structure of TsIIA with the groove in the DNA molecule, thus interfering with the synthesis and transcription of DNA and thus exerting the anti-tumor effect (53). This is consistent with our results. The cell composition enriched by GO shows that the target of TsIIA interferes with the normal assembly of various cell components, including cytoplasm, plasma membrane, nucleus, endoplasmic reticulum, etc. This further shows that the workplace of TsIIA is in the cell. In addition, the molecular function and biological process of GO enrichment also show that the target of TsIIA is involved in the activation and binding of a series of cell receptors and cascade downstream signal pathways, as well as in the positive regulation of cell proliferation, and regulates the activity of some protein kinases and the production of ATP. In addition, KEGG analysis showed that PI3K/Akt signaling pathway, MAPK signaling pathway, Ras signaling pathway and rap signaling pathway played a key role in the process of TsIIA anti HCC, which was consistent with previous studies. These pathways play an important role in the metastasis, progression, angiogenesis and energy metabolism of HCC. At the same time, in order to further find the common target of TsIIA regulating the appeal pathway, we extracted the intersection of TsIIA action targets enriched in the appeal pathway (**Figure 6**). We found that EGFR, Platelet-derived growth factor receptor B (PDGFRB), Platelet-derived growth factor receptor A (PDGFRA), Human mitogen activated protein kinase 1 (MAP2K1), Human mitogen activated protein kinase 2 (MAP2K2) and fibroblast growth factor receptor 3 (FGFR3) may be the key targets of TsIIA regulating the appeal pathway. It is worth mentioning that our results also show that TsIIA has a regulatory effect on microRNAs in HCC cells, and these small molecules also play a crucial role in tumor survival and metastasis. In addition, although some results were not within our screening range, they were statistically satisfactory (p < 0.05). For example, TsIIA also has a significant regulatory effect on the PD-1/PD-L1 signal pathway, the T-cell-related pathway, the immune helper cell, and related factors-mediated signal pathways. This shows that TsIIA also has a significant impact on the regulation of immune pathways. This is consistent with protein network analysis. In conclusion, the results of our network analysis and existing laboratory data show that TsIIA can inhibit the metastasis, invasion, and progression of HCC through multiple targets and pathways.

## 7 Molecular mechanism and target of TsIIA anti HCC

As we all know, the occurrence of tumors involves multiple pathways and multiple targets, and blocking a single signal pathway or gene product is usually not enough to prevent or treat malignant tumors. Previous studies have shown that the occurrence of HCC not only involves the abnormal expression and mutation of multiple oncogenes (p53, ras, myc and PTEN), but also is regulated by multiple abnormally activated signal pathways (PI3K-AKT, MAPK, JAK/STAT, NF-κB, mTOR and Wnt/β- catenin) (147). They jointly promote the occurrence of tumors and maintain the malignant behavior of tumor cells, tumor angiogenesis, tumor cell proliferation, metastasis and highly invasive formation (148–150). Through network pharmacological analysis, gene ontology (GO), Kyoto Encyclopedia of Genes and Genomes (KEGG) and review of a large number of previous studies, we found that TsIIA has extremely high targeting activity to these targets and pathways. In fact, TsIIA has been widely studied by scholars at home and abroad because of its special role in multi target and multi pathway. However, the pharmacological effect of TsIIA, which can inhibit the occurrence and development of HCC through multiple pathways and targets, is partly due to the significant antioxidant activity of TsIIA. Previous studies have confirmed that the overproduction of ROS in cells can not only directly lose DNA molecules and induce the occurrence and progression of tumors, but also significantly promote the metabolic reorganization of tumors and activate intracellular pathways related to tumor occurrence and progression. However, TsIIA can stabilize DNA molecules, down-regulate the activity of some pathways, and change the metabolic reorganization of tumor cells by inhibiting the level of ROS in cells and increasing antioxidant activity (53, 151, 152). Secondly, we have discussed the antioxidant activity of TsIIA in the section on liver protection in this article. In fact, different signal pathways can not only accept the activation of the same signal source but also have the same upstream and downstream molecules and even have obvious crosstalk.

**Figure 7:**
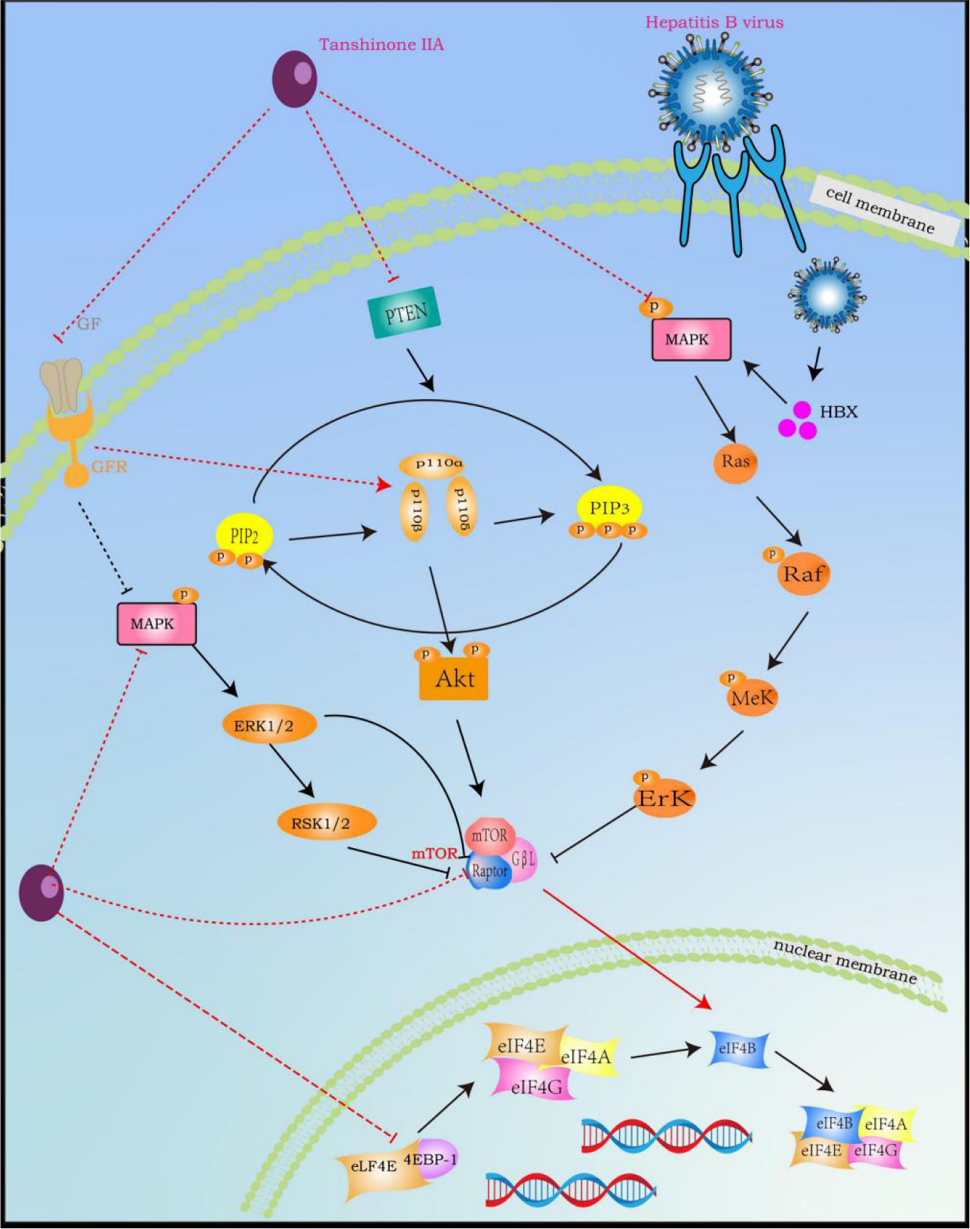
Tanshinone IIA Signal Pathway Interaction Process (induced by Tanshinone IIA are noted by using →, while the inhibition represented by ⊣ symbol). MAPK signal pathway is correlated with RAS and PI3K-AKT-mTOR. Tanshinone IIA can inhibit the occurrence, development, migration and vascularization of hepatocytes by blocking related pathways by inhibiting common upstream and downstream molecules, growth factors and their receptors.

Mitogen-activated protein kinase (MAPK) is a serine/threonine kinase that plays an important role in regulating cell activity and signal transduction, including cell proliferation, differentiation, survival, death, and transformation, and is closely related to the occurrence and transfer of HCC, especially in HCC caused by HBV infection, because HBV can encode a regulatory protein (HBx). This protein can activate some genes involved in cell proliferation regulation in the MAPK pathway, such as Ras, Raf, ERK, and JNK, to increase proliferation and inhibit apoptosis (153–155). However, TsIIA not only directly inhibits the expression and phosphorylation of MAPK (156), but also inhibits the activation and expression of related molecules in its mediated signal pathway, including ERK, JNK, p38 MAPK, etc. (157). In addition, it can also indirectly affect MAPK by up-regulating ROS in cancer cells (158). Secondly, the activation of the phosphatidylinositol 3-kinase (PI3K)/protein kinase B (AKT)/rapamycin (mTOR) pathway exists in 30%–50% of HCC (149). The up-regulated p-AKT and p-mTOR are also considered to be related to HCC grading, vascular invasion, and intrahepatic metastasis, so inhibiting this pathway is particularly important in the treatment of HCC (159, 160). However, it is worth noting that MAPK and mTOR-mediated signal pathways have mutual interference in many aspects. MAPK-related signal molecules can promote the activation of mTORC by phosphorylating the core component of mTOR (raptor). For example, MEK1/2 can not only phosphorylate raptor but also promote the phosphorylation of raptor through ERK1/2 and p90 ribosomal S6 kinase (RSK1/2), thereby increasing the activity of the mTORC pathway (155, 161). Secondly, MAPK can also regulate the activation of its upstream signal molecule PI3K through the small G proteins Ras and Raf kinase (155, 162). Therefore, TsIIA can indirectly inhibit the PI3K-AKT-mTOR pathway by inhibiting the MAPK pathway. In fact, TsIIA also has a significant inhibitory effect on the PI3K-AKT-mTOR signaling pathway. TsIIA can not only directly inhibit the expression and phosphorylation of mTOR to interfere with the activity of mTORC (163), but also inhibit the important upstream activator growth factor and its receptor for mTORC (164). Secondly, TsIIA can directly inhibit the protein expression and phosphorylation of PI3K and also inhibit the activation of this pathway by inhibiting the catalytic subunit p110α and regulatory subunit p85 of PI3K (165, 166). It is worth mentioning that TsIIA can inhibit the activity of the two pathways by inhibiting the same downstream molecules of MAPK and mTORC, such as Src kinase, FOXO (Fork Head Box O), c-Myc transcription factor, and various metabolically related enzymes (167, 168) (**Figure 7**).

Furthermore, numerous laboratory studies show that TsIIA can treat cancer by regulating the microRNA (miRNA) in tumor cells. It includes regulating the miR30b-p53 PTPN11/SHP2 pathway to induce the death of liver cancer cells (20), up-regulating the expression of miR-205 in ovarian cancer cells to induce apoptosis (169), targeting the metabolic regulation of miR-122/PKM2 to treat esophageal cancer, and inhibiting the expression level of microRNA-155 in colon cancer to inhibit colon cancer (170). This is also reflected in our KEGG enrichment analysis. MiRNA is a kind of endogenous, evolutionarily conservative, small single-stranded non-coding RNA (171). The length is 18–25 nucleotides, which contains more than 1000 miRNAs in the human genome, accounting for about 3% of the total number of human genes (172). It first generates hairpin primary miRNA in the nucleus through RNA polymerase and then generates precursor miRNA by Drosha enzyme cleavage. Subsequently, Exportin-5 transfers the precursor miRNA into the cytoplasm, where it is cleaved by the Dicer enzyme to produce mature miRNAs (173). MiRNA regulates gene expression and plays a crucial role in various biological processes, including development, cell cycle, apoptosis, differentiation, and angiogenesis (174). The imbalance in its expression is significant for the occurrence and progression of tumors. In addition, miRNA has been considered as a promising target for tumor therapy in previous studies. In fact, miRNA has been widely studied in the past decade as a key target for the occurrence and treatment of HCC through the regulation of epigenetic events. In addition, some studies have shown that HBV interferes with Wnt, MAPK and other signal pathways by regulating different microRNAs (miR-150, miR-342-3p, miR-375 and miR-25) to promote the occurrence of HCC (175). Secondly, miR-584, miR-517c and miR-378 have also been confirmed to enhance hepatocarcinogenesis if transferred from cancer cells to receptor normal cells (176, 177). Therefore, TsIIA should pay more attention to miRNA in liver cancer in future research.

## 8 The clinical application dilemma of tanshinone IIA

Since 1968, Ballie and Thomson et al. have fully synthesized TsIIA for the first time with 7-methoxy-1-tetrahydrozolidone as the starting material. The synthesis and clinical application of TsIIA and its derivatives have been widely discussed by scholars at home and abroad. Therefore, we also derived many synthetic routes for TsIIA, including substituted benzene synthesis, naphthalene derivative synthesis, Diels-Alder addition, and other synthetic routes (178). Secondly, we also found that TsIIA derivatives can be generated through a series of chemical reactions, including acylation, esterification, phosphorylation (179), sulfonation (180), bromination reaction (181), iodization reaction (182), oxidation reaction (183), and chloromethylation (184). However, these synthesis methods have the disadvantage of low output and high cost. Secondly, as far as the structure of TsIIA is concerned, even though TsIIA has a unique polycyclic phenylenediamine structure, make it has better fat solubility and a smaller size. Unfortunately, like most natural plant drugs, TsIIA is also characterized by poor water solubility, low polarity, and low oral bioavailability, which makes it unable to maximize its bioavailability in tissues (185). In addition, the side effects and cytotoxicity of TsIIA are also one of the keys to the difficulty in clinical application. Wang et al. found in their experiments that TsIIA would cause severe growth inhibition, developmental malformation, and cardiac toxicity in zebrafish embryos at high concentrations (186). In addition, TsIIA has the disadvantages of high fat solubility and a short half-life, which also hinder its clinical application. Secondly, the relatively low potency and poor drug properties of TsIIA also hinder the further clinical development of TsIIA (178, 187). However, we are gratified that with the in-depth study of TsIIA and the efforts disdained by scientists, We found that the chemical modification and loading technology of TsIIA can greatly improve its bioavailability and targeting delivery capability, which also makes it a key step in overcoming the clinical application problems of TsIIA.

## 9 TsIIA targeted delivery

Although the clinical application of TsIIA as an anti-tumor drug still has a long way to go, with the in-depth research of scientists, we also see the hope of solving this problem. Many derivatives related to TsIIA are also being prepared. Among them, STS is a water-soluble derivative of TsIIA, which has been used in the treatment of coronary heart disease, angina pectoris, etc. (188). After intravenous administration, STS can be rapidly distributed to many tissues, including the liver, kidney, lung, heart, and spleen. Unfortunately, its concentration in the liver decreased rapidly 30 minutes after administration (189). In addition, although STS, as a water-soluble derivative of TsIIA, is similar in structure to TsIIA, its effectiveness in some pharmacological actions is different from that of TsIIA, or even opposite (188). Therefore, scientists have made other efforts to better develop TsIIA as an anti-HCC drug.It is reported that the development of TsIIA intravenous fat emulsion (TsIIA LE) attracted scientists’ attention as early as 10 years ago. Chu T et al. developed the TsIIA-LE. Its formulation is composed of 0.05% (w/v) Tan IIA, 20% (w/v) soybean oil-MCT mixture (1:1, w/w), 1.2% (w/v) soybean lecithin, 0.3% (w/v) F68, and 2.2% (w/v) glycerol. A high pressure homogenization at 100 MPa for 3 cycles was selected as the optimal homogenization process. has not only been proven to have significant anti-tumor activity but also high safety and stability (36). Similarly, Ma et al. also had a similar effect by encapsulating T. anisaki into a microemulsion (ME) composed of phospholipids, ethyl oleate, glycerol, and Planck F68 (46).

In addition, the development of nanoparticle technology also provides a direction for the clinical transformation of TsIIA. Compared with TsIIA suspension, all kinds of nanoparticles containing TsIIA reported in the existing literature achieve higher concentration and longer retention time in the liver. These nano preparationinclude nano particles formed by encapsulating TsIIA into globins (TA Gb NPs),novel poly (lactic acid) nanoparticles containing TsIIA (TS PLA NPs) were synthesized by a single oil in water lotion/solvent evaporation method.The preparation formed by loading TsIIA into mPEG-PLGA-PLL-cRGD (methoxy polyethylene glycol, polylactic acid glycolic acid, poly L-lysine, cyclic arginine glycine aspartic acid) nanoparticles (TNP) (39, 44, 190). The latest nano preparation is a nano particle formed by Zhu et al. through packaging TsIIA into a drug carrier constructed by polyethylene imine (PEI) - polyethylene glycol (PEG) coated mesoporous silica nanoparticles (MSN-PEG). It not only has satisfactory efficacy, good dispersion, appropriate particle size and slow release effect, but also has significant transfection efficiency and DNA binding biological characteristics (27).In addition, the newly developed vesicle system targeting HCC also shows great prospects. The galactose modified PH sensitive Niosomes developed by Hu X et al. can not only target the liver, but also significantly prolong the blood circulation time of TsIIA, and also found that the strength of this ability is positively related to the content of galactose (156). It is worth mentioning that the existence of mixed micelles also enhances the possibility of developing TsIIA anti liver cancer drugs (30).

## 10 Conclusions and prospects

Up to now, The occurrence of cancer is often a process of multiple targets and pathways. Therefore, highly effective anticancer drugs often need to have multi pathway and multi target biological activities.However, TsIIA is expected to become a candidate drug for HCC treatment due to its ability to inhibit the proliferation, survival and migration of human HCC through multiple targets, links and pathways, and as a synergist of chemotherapy drugs. It can even change their drug resistance.Although TsIIA has been widely reported to be beneficial to health, lead to its clinical application is limited due to its low bioavailability. Fortunately, as described above, scientists are preparing microemulsions, microspheres, solid dispersions, liposomes, nanoparticles and other new dosage forms of TsIIA injection to significantly increase the drug concentration of TsIIA in HCC tissues and improve its bioavailability.However, in order to better understand the mechanism of action of TsIIA, improve the bioavailability, safety, dose effect and stability of TsIIA, and transform TsIIA into a candidate drug for treating HCC, further preclinical and clinical studies are needed in the future.

Secondly, the safety of the extraction of natural plants will also hinder the clinical application of drugs. Fortunately, the safety of TsIIA has been gradually confirmed. At present, the TsIIA aqueous solvent STS has been widely, safely, and effectively used in clinical practice in China. At the same time, in our review of the *in vivo* experiments of TsIIA and HCC, we did not find that TsIIA had toxic effects on other tissues except HCC. But these studies are based on *in vitro* cell experiments and *in vivo* animal experiments. Therefore, future research needs us to make clear the metabolism of TsIIA in human liver tissue, its side effects, and safety through well-designed human intervention experiments and pharmacokinetic experiments. In conclusion, all experimental data show that TsIIA can inhibit inflammatory reactions, oxidative stress, mediate apoptosis signal molecules, improve obesity and diabetes, promote stagnation of the hepatocyte cycle, and inhibit angiogenesis of hepatocytes *in vivo* and *in vitro*.These biological effects promote that TsIIA is expected to become a new drug or auxiliary drug for anti HCC.

## Author contributions

ZZ conceptualized the review, analyzed the data, and helped write the manuscript. HL, HP, YZ, XZ, XL, LY, and YX helped to write the manuscript and prepared the figures. All authors read and approved the final manuscript. All authors contributed to the article and approved the submitted version.

## References

[1] Yu XN Chen H Liu TT Wu J Zhu JM Shen XZ. Targeting the mTOR regulatory network in hepatocellular carcinoma: Are we making headway? Biochim Biophys Acta Rev Cancer (2019) 1871:379–91. doi: 10.1016/j.bbcan.2019.03.001 30951815

[2] Yang JD Hainaut P Gores GJ Amadou A Plymoth A Roberts LR. A global view of hepatocellular carcinoma: trends, risk, prevention and management. Nat Rev Gastroenterol Hepatol (2019) 16:589–604. doi: 10.1038/s41575-019-0186-y 31439937 PMC6813818

[3] Liu C Yang S Wang K Bao X Liu Y Zhou S. Alkaloids from traditional Chinese medicine against hepatocellular carcinoma. BioMed Pharmacother (2019) 120:109543. doi: 10.1016/j.biopha.2019.109543 31655311

[4] Kulik L El-Serag HB. Epidemiology and management of hepatocellular carcinoma. Gastroenterology (2019) 156:477–491.e1. doi: 10.1053/j.gastro.2018.08.065 30367835 PMC6340716

[5] Heimbach JK Kulik LM Finn RS Sirlin CB Abecassis MM Roberts LR. AASLD guidelines for the treatment of hepatocellular carcinoma. Hepatol (Baltimore Md.) (2018) 67:358–80. doi: 10.1002/hep.29086 28130846

[6] Gadsden MM Kaplan DE. Multidisciplinary approach to HCC management: How can this be done? Digest Dis Sci (2019) 64:968–75. doi: 10.1007/s10620-019-05593-8 30887152

[7] Forner A Reig M Bruix J. Hepatocellular carcinoma. Lancet (London England) (2018) 391:1301–14. doi: 10.1016/S0140-6736(18)30010-2 29307467

[8] Anwanwan D Singh SK Singh S Saikam V Singh R. Challenges in liver cancer and possible treatment approaches. Biochim Biophys Acta Rev Cancer (2020) 1873:188314. doi: 10.1016/j.bbcan.2019.188314 31682895 PMC6981221

[9] Tinkle CL Haas-Kogan D. Hepatocellular carcinoma: natural history, current management, and emerging tools. Biol: Targets Ther (2012) 6:207–19. doi: 10.2147/BTT.S23907 PMC3421475 22904613

[10] Chang Y Jeong SW Young Jang J Jae Kim Y. Recent updates of transarterial chemoembolilzation in hepatocellular carcinoma. Int J Mol Sci (2020) 21. doi: 10.3390/ijms21218165 PMC7662786 33142892

[11] Wu TC Shen YC Cheng AL. Evolution of systemic treatment for advanced hepatocellular carcinoma. Kaohsiung J Med Sci (2021) 37:643–53. doi: 10.1002/kjm2.12401 PMC11896262 34213069

[12] Yamamoto S Kondo S. Oral chemotherapy for the treatment of hepatocellular carcinoma. Expert Opin pharmacother (2018) 19:993–1001. doi: 10.1080/14656566.2018.1479398 29913090

[13] Pang H Wu L Tang Y Zhou G Qu C Duan JA. Chemical analysis of the herbal medicine salviae miltiorrhizae radix et rhizoma (Danshen). Molecules (2016) 21:51. doi: 10.3390/molecules21010051 26742026 PMC6273254

[14] Jiang Z Gao W Huang L. Tanshinones, critical pharmacological components in salvia miltiorrhiza. Front Pharmacol (2019) 10:202. doi: 10.3389/fphar.2019.00202 30923500 PMC6426754

[15] Kai G Xu H Zhou C Liao P Xiao J Luo X. Metabolic engineering tanshinone biosynthetic pathway in salvia miltiorrhiza hairy root cultures. Metab Eng (2011) 13:319–27. doi: 10.1016/j.ymben.2011.02.003 21335099

[16] Cao W Wang Y Shi M Hao X Zhao W Wang Y. Transcription factor SmWRKY1 positively promotes the biosynthesis of tanshinones in salvia miltiorrhiza. Front Plant Sci (2018) 9:554. doi: 10.3389/fpls.2018.00554 29755494 PMC5934499

[17] Lai Z He J Zhou C Zhao H Cui S. Tanshinones: An update in the medicinal chemistry in recent 5 years. Curr Med Chem (2021) 28:2807–27. doi: 10.2174/0929867327666200521124850 32436817

[18] Fang ZY Zhang M Liu JN Zhao X Zhang YQ Fang L. A review of its anticancer effects. Front Pharmacol (2020) 11:611087. doi: 10.3389/fphar.2020.611087 33597880 PMC7883641

[19] Rahman N Jeon M Song HY Kim YS. Cryptotanshinone, a compound of salvia miltiorrhiza inhibits pre-adipocytes differentiation by regulation of adipogenesis-related genes expression *via* STAT3 signaling. Phytomedicine (2016) 23:58–67. doi: 10.1016/j.phymed.2015.12.004 26902408

[20] Ren X Wang C Xie B Hu L Chai H Ding L. Tanshinone IIA induced cell death *via* miR30b-p53-PTPN11/SHP2 signaling pathway in human hepatocellular carcinoma cells. Eur J Pharmacol (2017) 796:233–41. doi: 10.1016/j.ejphar.2016.11.046 27894814

[21] Yuan SL Wei YQ Wang XJ Xiao F Li SF Zhang J. Growth inhibition and apoptosis induction of tanshinone II-a on human hepatocellular carcinoma cells. World J Gastroenterol (2004) 10:2024–8. doi: 10.3748/wjg.v10.i14.2024 PMC4572326 15237427

[22] Jiang T Zhu AS Yang CQ Xu CY Yang DQ Lou ZH. Cytochrome P450 2A6 is associated with macrophage polarization and is a potential biomarker for hepatocellular carcinoma. FEBS Open Bio (2021) 11:670–83. doi: 10.1002/2211-5463.13089 PMC7931228 33455085

[23] Lin CY Chang TW Hsieh WH Hung MC Lin IH Lai SC. Simultaneous induction of apoptosis and necroptosis by tanshinone IIA in human hepatocellular carcinoma HepG2 cells. Cell Death Discovery (2016) 2:16065. doi: 10.1038/cddiscovery.2016.65 27752362 PMC5045965

[24] Chen F Zhang J He Y Fang X Wang Y Chen M. Glycyrrhetinic acid-decorated and reduction-sensitive micelles to enhance the bioavailability and anti-hepatocellular carcinoma efficacy of tanshinone IIA. Biomater Sci (2016) 4:167–82. doi: 10.1039/C5BM00224A 26484363

[25] Long X Zhang J Zhang Y Yao J Cai Z Yang P. Nano-LC-MS/MS based proteomics of hepatocellular carcinoma cells compared to Chang liver cells and tanshinone IIA induction. Mol Biosyst (2011) 7:1728–41. doi: 10.1039/c0mb00343c 21423987

[26] Chang TW Lin CY Tzeng YJ Lur HS. Synergistic combinations of tanshinone IIA and trans-resveratrol toward cisplatin-comparable cytotoxicity in HepG2 human hepatocellular carcinoma cells. Anticancer Res (2014) 34:5473–80. 25275043

[27] Zhu Y Yue M Guo T Li F Li Z Yang D. PEI-PEG-Coated mesoporous silica nanoparticles enhance the antitumor activity of tanshinone IIA and serve as a gene transfer vector. Evidence-Based complement Altern med.: eCAM (2021) 2021:6756763. doi: 10.1155/2021/6756763 PMC8592735 34790248

[28] Liu Z Yan R Al-Salman A Shen Y Bu Y Ma J. Epidermal growth factor induces tumour marker AKR1B10 expression through activator protein-1 signalling in hepatocellular carcinoma cells. Biochem J (2012) 442:273–82. doi: 10.1042/BJ20111322 22329800

[29] Zhao L Xu J Jiao Y Wang H Fan S. Novel mechanisms involving chemically modified tetracycline 3 cytotoxicity. Anti-cancer Drugs (2014) 25:1165–74. doi: 10.1097/CAD.0000000000000144 27163120

[30] Zhang J Li Y Fang X Zhou D Wang Y Chen M. TPGS-g-PLGA/Pluronic F68 mixed micelles for tanshinone IIA delivery in cancer therapy. Int J pharmaceutics (2014) 476:185–98. doi: 10.1016/j.ijpharm.2014.09.017 25223472

[31] Yuxian X Feng T Ren L Zhengcai L. Tanshinone II-a inhibits invasion and metastasis of human hepatocellular carcinoma cells *in vitro* and in vivo. Tumori (2009) 95:789–95. doi: 10.1177/030089160909500623 20210245

[32] Cheng CY Su CC. Tanshinone IIA inhibits hep-J5 cells by increasing calreticulin, caspase 12 and GADD153 protein expression. Int J Mol Med (2010) 26:379–85. doi: 10.3892/ijmm_00000476 20664954

[33] Lee WY Cheung CC Liu KW Fung KP Wong J Lai PB. Cytotoxic effects of tanshinones from salvia miltiorrhiza on doxorubicin-resistant human liver cancer cells. J Natural prod. (2010) 73:854–9. doi: 10.1021/np900792p 20455578

[34] Zhong C Zhang YF Huang JH Wang ZY Chen QY Su LT. The Chinese medicine, jianpi huayu decoction, inhibits the epithelial mesenchymal transition *via* the regulation of the Smad3/Smad7 cascade. Am J Trans Res (2017) 9:2694–711. PMC5489874 28670362

[35] Dai ZK Qin JK Huang JE Luo Y Xu Q Zhao HL. Tanshinone IIA activates calcium-dependent apoptosis signaling pathway in human hepatoma cells. J Natural Medicines (2012) 66:192–201. doi: 10.1007/s11418-011-0576-0 21853384

[36] Chu T Zhang Q Li H Ma WC Zhang N Jin H. Development of intravenous lipid emulsion of tanshinone IIA and evaluation of its anti-hepatoma activity in vitro. Int J pharmaceutics (2012) 424:76–88. doi: 10.1016/j.ijpharm.2011.12.049 22226873

[37] Zhai XM He SX Ren MD Chen JH Wang ZL Han M. [Effect of tanshinone II a on expression of EGF and EGFR in hepatocellular carcinoma cell line SMMC-7721]. Zhejiang da xue xue bao Yi xue ban = J Zhejiang Univ Med Sci (2009) 38:163–9. doi: 10.3785/j.issn.1008-9292.2009.02.008 19363824

[38] Zhong ZH Chen WG Liu YH Li QX Qiu Y. [Inhibition of cell growth and induction of apoptosis in human hepatoma cell line HepG2 by tanshione IIA]. Zhong nan da xue xue bao Yi xue ban = J Cent South Univ Med Sci (2007) 32:99–103. 17344596

[39] Li Q Wang Y Feng N Fan Z Sun J Nan Y. Novel polymeric nanoparticles containing tanshinone IIA for the treatment of hepatoma. J Drug Targeting (2008) 16:725–32. doi: 10.1080/10611860802374303 19005937

[40] Liu TL Zhang LN Gu YY Lin MG Xie J Chen YL. The synergistic antitumor effect of tanshinone IIA plus adriamycin on human hepatocellular carcinoma xenograft in BALB/C nude mice and their influences on cytochrome P450 CYP3A4 in vivo. Adv Med (2020) 2020:6231751. doi: 10.1155/2020/6231751 34189145 PMC8192217

[41] Wang WQ Liu L Sun HC Fu YL Xu HX Chai ZT. Tanshinone IIA inhibits metastasis after palliative resection of hepatocellular carcinoma and prolongs survival in part *via* vascular normalization. J Hematol Oncol (2012) 5:69. doi: 10.1186/1756-8722-5-69 23137165 PMC3506473

[42] Ma H Fan Q Yu J Xin J Zhang C. Anticancer activities of tanshinone microemulsion against hepatocellular carcinoma in vitro and in vivo. Mol Med Rep (2013) 7:59–64. doi: 10.3892/mmr.2012.1129 23064251

[43] Chien SY Kuo SJ Chen YL Chen DR Cheng CY Su CC. Tanshinone IIA inhibits human hepatocellular carcinoma J5 cell growth by increasing bax and caspase 3 and decreasing CD31 expression in vivo. Mol Med Rep (2012) 5:282–6. doi: 10.3892/mmr.2011.631 22002472

[44] Wang Y Song D Costanza F Ji G Fan Z Cai J. Targeted delivery of tanshinone IIA-conjugated mPEG-PLGA-PLL-cRGD nanoparticles to hepatocellular carcinoma. J Biomed nanotechnol (2014) 10:3244–52. doi: 10.1166/jbn.2014.1982 26000384

[45] Jeon YJ Kim JS Hwang GH Wu Z Han HJ Park SH. Inhibition of cytochrome P450 2J2 by tanshinone IIA induces apoptotic cell death in hepatocellular carcinoma HepG2 cells. Eur J Pharmacol (2015) 764:480–8. doi: 10.1016/j.ejphar.2015.07.047 26209360

[46] Ma H Fan Q Yu J Xin J Zhang C. Novel microemulsion of tanshinone IIA, isolated from salvia miltiorrhiza bunge, exerts anticancer activity through inducing apoptosis in hepatoma cells. Am J Chin Med (2013) 41:197–210. doi: 10.1142/S0192415X13500146 23336516

[47] Khan HA Ahmad MZ Khan JA Arshad MI. Crosstalk of liver immune cells and cell death mechanisms in different murine models of liver injury and its clinical relevance. Hepatob pancreatic Dis int: HBPD Int (2017) 16:245–56. doi: 10.1016/S1499-3872(17)60014-6 PMC7172563 28603092

[48] Han H Desert R Das S Song Z Athavale D Ge X. Danger signals in liver injury and restoration of homeostasis. J Hepatol (2020) 73:933–51. doi: 10.1016/j.jhep.2020.04.033 PMC7502511 32371195

[49] Cichoż-Lach H Michalak A. Oxidative stress as a crucial factor in liver diseases. World J Gastroenterol (2014) 20:8082–91. doi: 10.3748/wjg.v20.i25.8082 PMC4081679 25009380

[50] Yang S Lian G. ROS and diseases: role in metabolism and energy supply. Mol Cell Biochem (2020) 467:1–12. doi: 10.1007/s11010-019-03667-9 31813106 PMC7089381

[51] Elias-Miró M Jiménez-Castro MB Rodés J Peralta C. Current knowledge on oxidative stress in hepatic ischemia/reperfusion. Free Radical Res (2013) 47:555–68. doi: 10.3109/10715762.2013.811721 23738581

[52] Koyama Y Brenner DA. Liver inflammation and fibrosis. J Clin Invest (2017) 127:55–64. doi: 10.1172/JCI88881 28045404 PMC5199698

[53] Li W Huang T Xu S Che B Yu Y Zhang W. Molecular mechanism of tanshinone against prostate cancer. Molecules (2022) 27. doi: 10.3390/molecules27175594 PMC9457553 36080361

[54] Lu TC Wu YH Chen WY Hung YC. Targeting oxidative stress and endothelial dysfunction using tanshinone IIA for the treatment of tissue inflammation and fibrosis. Oxid Med Cell Longevity (2022) 2022:2811789. doi: 10.1155/2022/2811789 PMC9010204 35432718

[55] Zhong C Lin Z Ke L Shi P Li S Huang L. Recent research progress (2015-2021) and perspectives on the pharmacological effects and mechanisms of tanshinone IIA. Front Pharmacol (2021) 12:778847. doi: 10.3389/fphar.2021.778847 34819867 PMC8606659

[56] He L Liu YY Wang K Li C Zhang W Li ZZ. Tanshinone IIA protects human coronary artery endothelial cells from ferroptosis by activating the NRF2 pathway. Biochem Biophys Res Commun (2021) 575:1–7. doi: 10.1016/j.bbrc.2021.08.067 34454174

[57] Brenner C Galluzzi L Kepp O Kroemer G. Decoding cell death signals in liver inflammation. J Hepatol (2013) 59:583–94. doi: 10.1016/j.jhep.2013.03.033 23567086

[58] Oeckinghaus A Ghosh S. The NF-kappaB family of transcription factors and its regulation. Cold Spring Harbor Perspect Biol (2009) 1:a000034. doi: 10.1101/cshperspect.a000034 PMC2773619 20066092

[59] Tiegs G Horst AK. TNF in the liver: targeting a central player in inflammation. Semin immunopathol (2022) 44:445–59. doi: 10.1007/s00281-022-00910-2 PMC9256556 35122118

[60] He G Karin M. NF-κB and STAT3 - key players in liver inflammation and cancer. Cell Res (2011) 21:159–68. doi: 10.1038/cr.2010.183 PMC3193410 21187858

[61] Xu F Liu C Zhou D Zhang L. TGF-β/SMAD pathway and its regulation in hepatic fibrosis. J Histochem Cytochem (2016) 64:157–67. doi: 10.1369/0022155415627681+ PMC4810800 26747705

[62] Wang X Guo D Li W Zhang Q Jiang Y Wang Q. Danshen (Salvia miltiorrhiza) restricts MD2/TLR4-MyD88 complex formation and signalling in acute myocardial infarction-induced heart failure. J Cell Mol Med (2020) 24:10677–92. doi: 10.1111/jcmm.15688 PMC7521313 32757377

[63] Gao H Liu X Sun W Kang N Liu Y Yang S. Total tanshinones exhibits anti-inflammatory effects through blocking TLR4 dimerization *via* the MyD88 pathway. Cell Death Dis (2017) 8:e3004. doi: 10.1038/cddis.2017.389 PMC5596575 28817116

[64] Yang L Zhou G Liu J Song J Zhang Z Huang Q. And tanshinone IIA/B attenuate LPS-induced mastitis *via* regulating the NF-κB. BioMed Pharmacother (2021) 137:111353. doi: 10.1016/j.biopha.2021.111353 33578236

[65] Shan Z Ju C. Hepatic macrophages in liver injury. Front Immunol (2020) 11:322. doi: 10.3389/fimmu.2020.00322 32362892 PMC7180226

[66] Dar WA Sullivan E Bynon JS Eltzschig H Ju C. Ischaemia reperfusion injury in liver transplantation: Cellular and molecular mechanisms. Liver Int (2019) 39:788–801. doi: 10.1111/liv.14091 30843314 PMC6483869

[67] Qi YY Xiao L Zhang LD Song SH Mei Y Chen T. Tanshinone IIA pretreatment attenuates hepatic ischemia-reperfusion. Front biosci. (Elite edition) (2012) 4:1303–13. doi: 10.2741/e461 22201956

[68] Li X Wu Y Zhang W Gong J Cheng Y. Pre-conditioning with tanshinone IIA attenuates the ischemia/reperfusion injury caused by liver grafts *via* regulation of HMGB1 in rat kupffer cells. BioMed Pharmacother (2017) 89:1392–400. doi: 10.1016/j.biopha.2017.03.022 28320107

[69] Wang Y Ni Q Ye Q Liu F Fu Z Wang Q. Tanshinone IIA activates autophagy to reduce liver ischemia-reperfusion injury by MEK/ERK/mTOR pathway. Die Pharmazie (2018) 73:396–401. doi: 10.1691/ph.2018.7509 30001774

[70] Shi MJ Dong BS Yang WN Su SB Zhang H. Preventive and therapeutic role of tanshinone II A in hepatology. BioMed Pharmacother (2019) 112:108676. doi: 10.1016/j.biopha.2019.108676 30797157

[71] Farghali H Kgalalelo Kemelo M Wojnarová L Kutinová Canová N. *In vitro* and *in vivo* experimental hepatotoxic models in liver research: applications to the assessment of potential hepatoprotective drugs. Physiol Res (2016) 65:S417–s425. doi: 10.33549/physiolres.933506 28006924

[72] Park EJ Zhao YZ Kim YC Sohn DH. Preventive effects of a purified extract isolated from salvia miltiorrhiza enriched with tanshinone I, tanshinone IIA and cryptotanshinone on hepatocyte injury *in vitro* and in vivo. Food Chem Toxicol (2009) 47:2742–8. doi: 10.1016/j.fct.2009.08.007 19695300

[73] Wang W Guan C Sun X Zhao Z Li J Fu X. Tanshinone IIA protects against acetaminophen-induced hepatotoxicity *via* activating the Nrf2 pathway. Phytomedicine (2016) 23:589–96. doi: 10.1016/j.phymed.2016.02.022 27161400

[74] Guan CW Jin J Li J Zhao ZX Huang ZY. [Tanshinone IIA protects against triptolide-induced liver injury *via* Nrf2/ARE activation]. Yao xue xue bao = Acta Pharm Sin (2013) 48:1397–402. 24358772

[75] Qin XY Li T Yan L Liu QS Tian Y. Tanshinone IIA protects against immune-mediated liver injury through activation of T-cell subsets and regulation of cytokines. Immunopharmacol immunotoxicol (2010) 32:51–5. doi: 10.3109/08923970903120997 19653860

[76] Qian QY Ying N Yang Z Zhou L Liu QS Hu ZY. [Mechanisms of tanshinone II_A in reducing 4-HNE-induced hepatocyte damage by activating PPARα]. Zhongguo Zhong yao za zhi = Zhongguo zhongyao zazhi = China J Chin mater Med (2019) 44:1862–8. doi. 10.19540/j.cnki.cjcmm.20190305.002 31342714

[77] Yue S Hu B Wang Z Yue Z Wang F Zhao Y. Salvia miltiorrhiza compounds protect the liver from acute injury by regulation of p38 and NFκB signaling in kupffer cells. Pharm Biol (2014) 52:1278–85. doi: 10.3109/13880209.2014.889720 25026357

[78] Huang W Dong Z Wei H Ding C Sun R Tian Z. Selective elimination of hepatic natural killer T cells with concanavalin a improves liver regeneration in mice. Liver int: Off J Int Assoc Study Liver (2006) 26:339–45. doi: 10.1111/j.1478-3231.2005.01221.x 16584397

[79] Irshad M Khushboo I Singh S Singh S. Hepatitis c virus (HCV): a review of immunological aspects. Int Rev Immunol (2008) 27:497–517. doi: 10.1080/08830180802432178 19065353

[80] Lu L Zhou J Zhang J Che J Jiao Y Zhang Y. Prevention and therapeutic effects and mechanisms of tanshinone IIA sodium sulfonate on acute liver injury mice model. Evidence-Based complement Altern med.: eCAM (2016) 2016:4097398. doi. 10.1155/2016/4097398 PMC4870345 27274751

[81] Ma S Wang X Wang Y Zuo X. Sodium tanshinone IIA sulfonate improves hemodynamic parameters, cytokine release, and multi-organ damage in endotoxemia rabbits. Med Sci monitor: Int Med J Exp Clin Res (2018) 24:2975–82. doi: 10.12659/MSM.909996 PMC5968839 29735976

[82] Xu Y Feng D Wang Y Lin S Xu L. Sodium tanshinone IIA sulfonate protects mice from ConA-induced hepatitis *via* inhibiting NF-kappaB and IFN-gamma/STAT1 pathways. J Clin Immunol (2008) 28:512–9. doi: 10.1007/s10875-008-9206-3 18498044

[83] Zhang CY Yuan WG He P Lei JH Wang CX. Liver fibrosis and hepatic stellate cells: Etiology, pathological hallmarks and therapeutic targets. World J Gastroenterol (2016) 22:10512–22. doi: 10.3748/wjg.v22.i48.10512 PMC5192262 28082803

[84] Aydın MM Akçalı KC. Liver fibrosis. Turkish J Gastroenterol (2018) 29:14–21. doi: 10.5152/tjg.2018.17330 PMC6322608 29391303

[85] Sun X Tan Y Lyu J Liu HL Zhao ZM Liu CH. Active components formulation developed from fuzheng huayu recipe for anti-liver fibrosis. Chin J Integr Med (2022) 28:538–44. doi: 10.1007/s11655-021-3293-x 34581939

[86] Higashi T Friedman SL Hoshida Y. Hepatic stellate cells as key target in liver fibrosis. Advanced Drug del Rev (2017) 121:27–42. doi: 10.1016/j.addr.2017.05.007 PMC5682243 28506744

[87] Pan TL Wang PW. Explore the molecular mechanism of apoptosis induced by tanshinone IIA on activated rat hepatic stellate cells. Evidence-Based complement Altern med.: eCAM (2012) 2012:734987. doi: 10.1155/2012/734987 PMC3546466 23346212

[88] Liu Y Chen H Jiang Y. [Effect of tanshinone IIA on CCl4-induced liver fibrosis in rats]. Zhong yao cai = Zhongyaocai = J Chin med mater (2002) 25:31–3. 12583240

[89] Che XH Park EJ Zhao YZ Kim WH Sohn DH. Tanshinone II a induces apoptosis and s phase cell cycle arrest in activated rat hepatic stellate cells. Basic Clin Pharmacol Toxicol (2010) 106:30–7. doi: 10.1111/j.1742-7843.2009.00465.x 19906051

[90] Sun RF Liu LX Zhang HY. [Effect of tanshinone II on hepatic fibrosis in mice]. Zhongguo Zhong xi yi jie he za zhi Zhongguo Zhongxiyi jiehe zazhi = Chin J integr tradit Western Med (2009) 29:1012–7. 20329614

[91] Wu R Dong S Cai FF Chen XL Yang MD Liu P. Active compounds derived from fuzheng huayu formula protect hepatic parenchymal cells from apoptosis based on network pharmacology and transcriptomic analysis. Molecules (2019) 24. doi: 10.3390/molecules24020338 PMC6358846 30669350

[92] Liu YW Huang YT. Inhibitory effect of tanshinone IIA on rat hepatic stellate cells. PloS One (2014) 9:e103229. doi: 10.1371/journal.pone.0103229 25076488 PMC4116159

[93] Ko S Russell JO Molina LM Monga SP. Liver progenitors and adult cell plasticity in hepatic injury and repair: Knowns and unknowns. Annu Rev Pathol (2020) 15:23–50. doi: 10.1146/annurev-pathmechdis-012419-032824 31399003 PMC7212705

[94] Chen J Chen L Zern MA Theise ND Diehl AM Liu P. The diversity and plasticity of adult hepatic progenitor cells and their niche. Liver Int (2017) 37:1260–71. doi: 10.1111/liv.13377 PMC5534384 28135758

[95] Ze X Jia J Li X You H Zhao X Zhang D. Tanshinone IIA promotes the proliferation of WB-F344 hepatic oval cells *via* wnt/β-catenin signaling. Mol Med Rep (2016) 13:1501–8. doi: 10.3892/mmr.2015.4696 PMC4732833 26709094

[96] Yang N Chen H Gao Y Zhang S Lin Q Ji X. Tanshinone IIA exerts therapeutic effects by acting on endogenous stem cells in rats with liver cirrhosis. BioMed Pharmacother (2020) 132:110815. doi: 10.1016/j.biopha.2020.110815 33113421

[97] Li T Apte U. Bile acid metabolism and signaling in cholestasis, inflammation, and cancer. Adv Pharmacol (San Diego Calif.) (2015) 74:263–302. doi: 10.1016/b.apha.2015.04.003 PMC4615692 26233910

[98] Stieger B. The role of the sodium-taurocholate cotransporting polypeptide (NTCP) and of the bile salt export pump (BSEP) in physiology and pathophysiology of bile formation. Handb Exp Pharmacol (2011) 201:205–59. doi: 10.1007/978-3-642-14541-4_5 21103971

[99] Yang Y Liu L Xu M Zhang X Wang L He Q. Tanshinone II A may alleviate rifampin-induced cholestasis by regulating the expression and function of NTCP. Hum Exp Toxicol (2021) 40:1003–11. doi: 10.1177/0960327120979030 33307820

[100] Zhang X Ma Z Liang Q Tang X Hu D Liu C. Tanshinone IIA exerts protective effects in a LCA-induced cholestatic liver model associated with participation of pregnane X receptor. J ethnopharmacol (2015) 164:357–67. doi: 10.1016/j.jep.2015.01.047 25660334

[101] Niu XH Hua HY Guo WJ Zhang Y Liu M Hong Y. [Clinical efficiency of tanshinone IIA-sulfonate in treatment of liver fibrosis of advanced schistosomiasis]. Zhongguo xue xi chong bing fang zhi za zhi = Chin J schistosomiasis control (2013) 25:137–40. 23894832

[102] Maurice J Manousou P. Non-alcoholic fatty liver disease. Clin Med (London England) (2018) 18:245–50. doi: 10.7861/clinmedicine.18-3-245 PMC6334080 29858436

[103] Manne V Handa P Kowdley KV. Pathophysiology of nonalcoholic fatty liver Disease/Nonalcoholic steatohepatitis. Clinics liver Dis (2018) 22:23–37. doi: 10.1016/j.cld.2017.08.007 29128059

[104] Pierantonelli I Svegliati-Baroni G. Nonalcoholic fatty liver disease: Basic pathogenetic mechanisms in the progression from NAFLD to NASH. Transplantation (2019) 103:e1–e13. doi: 10.1097/TP.0000000000002480 30300287

[105] Huang L Ding W Wang MQ Wang ZG Chen HH Chen W. Tanshinone IIA ameliorates non-alcoholic fatty liver disease through targeting peroxisome proliferator-activated receptor gamma and toll-like receptor 4. J Int Med Res (2019) 47:5239–55. doi: 10.1177/0300060519859750 PMC6833399 31378113

[106] Yang GL Jia LQ Wu J Ma YX Cao HM Song N. Effect of tanshinone IIA on oxidative stress and apoptosis in a rat model of fatty liver. Exp Ther Med (2017) 14:4639–46. doi: 10.3892/etm.2017.5162 PMC5704301 29201162

[107] Song HY Zhang L Pan JL Yang LL Ji G. Bioactivity of five components of Chinese herbal formula jiangzhi granules against hepatocellular steatosis. J Integr Med (2013) 11:262–8. doi: 10.3736/jintegrmed2013034 23867244

[108] Hong M Li S Wang N Tan HY Cheung F Feng Y. A biomedical investigation of the hepatoprotective effect of radix salviae miltiorrhizae and network pharmacology-based prediction of the active compounds and molecular targets. Int J Mol Sci (2017) 18. doi: 10.3390/ijms18030620 PMC5372635 28335383

[109] Li XX Lu XY Zhang SJ Chiu AP Lo LH Largaespada DA. Sodium tanshinone IIA sulfonate ameliorates hepatic steatosis by inhibiting lipogenesis and inflammation. BioMed Pharmacother (2019) 111:68–75. doi: 10.1016/j.biopha.2018.12.019 30576936

[110] Wang J Hu R Yin C Xiao Y. Tanshinone IIA reduces palmitate-induced apoptosis *via* inhibition of endoplasmic reticulum stress in HepG2 liver cells. Fundam Clin Pharmacol (2020) 34:249–62. doi: 10.1111/fcp.12510 31520549

[111] Yin HQ Kim YS Choi YJ Kim YC Sohn DH Ryu SY. Effects of tanshinone IIA on the hepatotoxicity and gene expression involved in alcoholic liver disease. Arch pharmacal Res (2008) 31:659–65. doi: 10.1007/s12272-001-1209-2 18481025

[112] Powell EE Wong VW Rinella M. Non-alcoholic fatty liver disease. Lancet (London England) (2021) 397:2212–24. doi: 10.1016/S0140-6736(20)32511-3 33894145

[113] Chalasani N Younossi Z Lavine JE Diehl AM Brunt EM Cusi K. The diagnosis and management of non-alcoholic fatty liver disease: practice guideline by the American association for the study of liver diseases, American college of gastroenterology, and the American gastroenterological association. Hepatol (Baltimore Md.) (2012) 55:2005–23. doi: 10.1002/hep.25762 22488764

[114] Dietrich P Hellerbrand C. Non-alcoholic fatty liver disease, obesity and the metabolic syndrome. Best Pract Res Clin Gastroenterol (2014) 28:637–53. doi: 10.1016/j.bpg.2014.07.008 25194181

[115] Bosserhoff A Hellerbrand C. Obesity and fatty liver are ‘grease’ for the machinery of hepatic fibrosis. Digest Dis (Basel Switzerland) (2011) 29:377–83. doi: 10.1159/000329800 21894008

[116] Yki-Järvinen H. Non-alcoholic fatty liver disease as a cause and a consequence of metabolic syndrome. Lancet Diabetes Endocrinol (2014) 2:901–10. doi: 10.1016/S2213-8587(14)70032-4 24731669

[117] Gaggini M Morelli M Buzzigoli E DeFronzo RA Bugianesi E Gastaldelli A. Non-alcoholic fatty liver disease (NAFLD) and its connection with insulin resistance, dyslipidemia, atherosclerosis and coronary heart disease. Nutrients (2013) 5:1544–60. doi: 10.3390/nu5051544 PMC3708335 23666091

[118] Xie Z Loi Truong T Zhang P Xu F Xu X Li P. Dan-Qi prescription ameliorates insulin resistance through overall corrective regulation of glucose and fat metabolism. J ethnopharmacol (2015) 172:70–9. doi: 10.1016/j.jep.2015.05.041 26087232

[119] Hwang SL Yang JH Jeong YT Kim YD Li X Lu Y. Tanshinone IIA improves endoplasmic reticulum stress-induced insulin resistance through AMP-activated protein kinase. Biochem Biophys Res Commun (2013) 430:1246–52. doi: 10.1016/j.bbrc.2012.12.066 23266607

[120] Yuan FY Zhang M Xu P Xu D Chen P Ren M. Tanshinone IIA improves diabetes mellitus *via* the NF-κB-induced AMPK signal pathway. Exp Ther Med (2018) 16:4225–31. doi: 10.3892/etm.2018.6674 PMC6176167 30344697

[121] Pistritto G Trisciuoglio D Ceci C Garufi A D’Orazi G. Apoptosis as anticancer mechanism: function and dysfunction of its modulators and targeted therapeutic strategies. Aging (2016) 8:603–19. doi: 10.18632/aging.100934 PMC4925817 27019364

[122] Goldar S Khaniani MS Derakhshan SM Baradaran B. Molecular mechanisms of apoptosis and roles in cancer development and treatment. Asian Pac J Cancer prev: APJCP (2015) 16:2129–44. doi: 10.7314/APJCP.2015.16.6.2129 25824729

[123] Bates S Bonetta L MacAllan D Parry D Holder A Dickson C. CDK6 (PLSTIRE) and CDK4 (PSK-J3) are a distinct subset of the cyclin-dependent kinases that associate with cyclin D1. Oncogene (1994) 9:71–9. 8302605

[124] Kato J Matsushime H Hiebert SW Ewen ME Sherr CJ. Direct binding of cyclin d to the retinoblastoma gene product (pRb) and pRb phosphorylation by the cyclin d-dependent kinase CDK4. Genes Dev (1993) 7:331–42. doi: 10.1101/gad.7.3.331 8449399

[125] Hernández-Monge J Rousset-Roman AB Medina-Medina I Olivares-Illana V. Dual function of MDM2 and MDMX toward the tumor suppressors p53 and RB. Genes Cancer (2016) 7:278–87. doi: 10.18632/genesandcancer.120 PMC5115168 28050229

[126] Lin C Wang L Wang H Yang L Guo H Wang X. Tanshinone IIA inhibits breast cancer stem cells growth *in vitro* and *in vivo* through attenuation of IL-6/STAT3/NF-kB signaling pathways. J Cell Biochem (2013) 114:2061–70. doi: 10.1002/jcb.24553 23553622

[127] Li Z Zhang Y Zhou Y Wang F Yin C Ding L. Tanshinone IIA suppresses the progression of lung adenocarcinoma through regulating CCNA2-CDK2 complex and AURKA/PLK1 pathway. Sci Rep (2021) 11:23681. doi: 10.1038/s41598-021-03166-2 34880385 PMC8654884

[128] Lambert AW Pattabiraman DR Weinberg RA. Emerging biological principles of metastasis. Cell (2017) 168:670–91. doi: 10.1016/j.cell.2016.11.037 PMC5308465 28187288

[129] Erdogan B Webb DJ. Cancer-associated fibroblasts modulate growth factor signaling and extracellular matrix remodeling to regulate tumor metastasis. Biochem Soc Trans (2017) 45:229–36. doi: 10.1042/BST20160387 PMC5371349 28202677

[130] Zhang Y Weinberg RA. Epithelial-to-mesenchymal transition in cancer: complexity and opportunities. Front Med (2018) 12:361–73. doi: 10.1007/s11684-018-0656-6 PMC6186394 30043221

[131] Lamouille S Xu J Derynck R. Molecular mechanisms of epithelial-mesenchymal transition. Nat Rev Mol Cell Biol (2014) 15:178–96. doi: 10.1038/nrm3758 PMC4240281 24556840

[132] Ma L Jiang H Xu X Zhang C Niu Y Wang Z. Tanshinone IIA mediates SMAD7-YAP interaction to inhibit liver cancer growth by inactivating the transforming growth factor beta signaling pathway. Aging (2019) 11:9719–37. doi: 10.18632/aging.102420 PMC6874425 31711043

[133] Qian SK Chen D Li Y Yang XW Deng WJ Li Q. [Effects of e-selectin and their ligands on the adhesive metastasis of hepatocellular carcinoma]. Zhonghua gan zang bing za zhi = Zhonghua ganzangbing zazhi = Chin J Hepatol (2010) 18:440–4. doi: 10.3760/cma.j.issn.1007-3418.2010.06.011 20587315

[134] Chiu CM Huang SY Chang SF Liao KF Chiu SC. Synergistic antitumor effects of tanshinone IIA and sorafenib or its derivative SC-1 in hepatocellular carcinoma cells. OncoTargets Ther (2018) 11:1777–85. doi: 10.2147/OTT.S161534 PMC5881525 29636623

[135] Zhang Y Tie M Wang K Bi F. Tanshinone II improves distribution and anti-tumor efficacy of pegylated liposomal doxorubicin *via* normalizing the structure and function of tumor vasculature in hepa1-6 hepatoma mice model. J tradit Chin Med = Chung i tsa chih ying wen pan (2018) 38:815–22. 32186128

[136] Hou LL Xu QJ Hu GQ Xie SQ. [Synergistic antitumor effects of tanshinone II a in combination with cisplatin *via* apoptosis in the prostate cancer cells]. Yao xue xue bao = Acta Pharm Sin (2013) 48:675–9. 23888689

[137] Jung JH Kwon TR Jeong SJ Kim EO Sohn EJ Yun M. Apoptosis induced by tanshinone IIA and cryptotanshinone is mediated by distinct JAK/STAT3/5 and SHP1/2 signaling in chronic myeloid leukemia K562 cells. Evidence-Based complement Altern med.: eCAM (2013) 2013:805639. doi: 10.1155/2013/805639 PMC3710643 23878608

[138] Su CC. Tanshinone IIA potentiates the efficacy of 5-FU in Colo205 colon cancer cells *in vivo* through downregulation of p-gp and LC3-II. Exp Ther Med (2012) 3:555–9. doi: 10.3892/etm.2011.441 PMC3438563 22969929

[139] Zhou SF Liu JP Chowbay B. Polymorphism of human cytochrome P450 enzymes and its clinical impact. Drug Metab Rev (2009) 41:89–295. doi: 10.1080/03602530902843483 19514967

[140] Nagai K Yoshida N Kiyama M Kasahara K Yamamura A Konishi H. Decreased elimination clearance of midazolam by doxorubicin through reductions in the metabolic activity of hepatic CYP3A in rats. Xenobiotica; fate foreign compd Biol Syst (2015) 45:874–80. doi: 10.3109/00498254.2015.1027971 26053556

[141] Ashida R Okamura Y Ohshima K Kakuda Y Uesaka K Sugiura T. CYP3A4 gene is a novel biomarker for predicting a poor prognosis in hepatocellular carcinoma. Cancer Genomics Proteomics (2017) 14:445–53. doi: 10.21873/cgp.20054 PMC6070324 29109094

[142] Ullah MF. Cancer multidrug resistance (MDR): a major impediment to effective chemotherapy. Asian Pac J Cancer prev: APJCP (2008) 9:1–6. 18439063

[143] Li K Liu W Zhao Q Wu C Fan C Lai H. Combination of tanshinone IIA and doxorubicin possesses synergism and attenuation effects on doxorubicin in the treatment of breast cancer. Phytother res: PTR (2019) 33:1658–69. doi: 10.1002/ptr.6353 30945389

[144] Zou S Tong Q Liu B Huang W Tian Y Fu X. Targeting STAT3 in cancer immunotherapy. Mol Cancer (2020) 19:145. doi: 10.1186/s12943-020-01258-7 32972405 PMC7513516

[145] Xu J Lin H Wu G Zhu M Li M. IL-6/STAT3 is a promising therapeutic target for hepatocellular carcinoma. Front Oncol (2021) 11:760971. doi: 10.3389/fonc.2021.760971 34976809 PMC8714735

[146] Venook AP Papandreou C Furuse J de Guevara LL. The incidence and epidemiology of hepatocellular carcinoma: a global and regional perspective. Oncologist (2010) 15(Suppl 4):5–13. doi: 10.1634/theoncologist.2010-S4-05 21115576

[147] Ramakrishna G Rastogi A Trehanpati N Sen B Khosla R Sarin SK. From cirrhosis to hepatocellular carcinoma: new molecular insights on inflammation and cellular senescence. Liver Cancer (2013) 2:367–83. doi: 10.1159/000343852 PMC3881319 24400224

[148] Alqahtani A Khan Z Alloghbi A Said Ahmed TS Ashraf M Hammouda DM. Hepatocellular carcinoma: Molecular mechanisms and targeted therapies. Med (Kaunas) (2019) 55. doi: 10.3390/medicina55090526 PMC6780754 31450841

[149] Ogunwobi OO Harricharran T Huaman J Galuza A Odumuwagun O Tan Y. Mechanisms of hepatocellular carcinoma progression. World J Gastroenterol (2019) 25:2279–93. doi: 10.3748/wjg.v25.i19.2279 PMC6529884 31148900

[150] Jelic MD Mandic AD Maricic SM Srdjenovic BU. Oxidative stress and its role in cancer. J Cancer Res Ther (2021) 17:22–8. doi: 10.4103/jcrt.JCRT_862_16 33723127

[151] Klaunig JE. Oxidative stress and cancer. Curr Pharm Des (2018) 24:4771–8. doi: 10.2174/1381612825666190215121712 30767733

[152] Janku F Kaseb AO Tsimberidou AM Wolff RA Kurzrock R. Identification of novel therapeutic targets in the PI3K/AKT/mTOR pathway in hepatocellular carcinoma using targeted next generation sequencing. Oncotarget (2014) 5:3012–22. doi: 10.18632/oncotarget.1687 PMC4102787 24931142

[153] Guo YJ Pan WW Liu SB Shen ZF Xu Y Hu LL. ERK/MAPK signalling pathway and tumorigenesis. Exp Ther Med (2020) 19:1997–2007. doi: 10.3892/etm.2020.8454 32104259 PMC7027163

[154] Carriere A Romeo Y Acosta-Jaquez HA Moreau J Bonneil E Thibault P. ERK1/2 phosphorylate raptor to promote ras-dependent activation of mTOR complex 1 (mTORC1). J Biol Chem (2011) 286:567–77. doi: 10.1074/jbc.M110.159046 PMC3013016 21071439

[155] Hu X Zhang J Deng L Hu H Hu J Zheng G. Galactose-modified PH-sensitive niosomes for controlled release and hepatocellular carcinoma target delivery of tanshinone IIA. AAPS PharmSciTech (2021) 22:96. doi: 10.1208/s12249-021-01973-4 33694067 PMC7946689

[156] Sun S Zhu L Lai M Cheng R Ge Y. Tanshinone I inhibited growth of human chronic myeloid leukemia cells *via* JNK/ERK mediated apoptotic pathways. Braz J Med Biol Res = Rev Bras pesquisas med e biol (2021) 54:e10685. doi: 10.1590/1414-431x2020e10685 PMC8148979 34037092

[157] Su CC. Tanshinone IIA can inhibit MiaPaCa-2 human pancreatic cancer cells by dual blockade of the Ras/Raf/MEK/ERK and PI3K/AKT/mTOR pathways. Oncol Rep (2018) 40:3102–11. doi: 10.3892/or.2018.6670 30226540

[158] Chen C Wang G. Mechanisms of hepatocellular carcinoma and challenges and opportunities for molecular targeted therapy. World J Hepatol (2015) 7:1964–70. doi: 10.4254/wjh.v7.i15.1964 PMC4517155 26244070

[159] Kudo M. Signaling pathway and molecular-targeted therapy for hepatocellular carcinoma. Digest Dis (Basel Switzerland) (2011) 29:289–302. doi: 10.1159/000327562 21829020

[160] Tambe Y Terado T Kim CJ Mukaisho KI Yoshida S Sugihara H. Antitumor activity of potent pyruvate dehydrogenase kinase 4 inhibitors from plants in pancreatic cancer. Mol carcinog (2019) 58:1726–37. doi: 10.1002/mc.23045 31106493

[161] Carrière A Cargnello M Julien LA Gao H Bonneil E Thibault P. Oncogenic MAPK signaling stimulates mTORC1 activity by promoting RSK-mediated raptor phosphorylation. Curr biol: CB (2008) 18:1269–77. doi: 10.1016/j.cub.2008.07.078 18722121

[162] Fu L Han B Zhou Y Ren J Cao W Patel G. The anticancer properties of tanshinones and the pharmacological effects of their active ingredients. Front Pharmacol (2020) 11:193. doi: 10.3389/fphar.2020.00193 32265690 PMC7098175

[163] Tee AR Manning BD Roux PP Cantley LC Blenis J. Tuberous sclerosis complex gene products, tuberin and hamartin, control mTOR signaling by acting as a GTPase-activating protein complex toward rheb. Curr biol: CB (2022) 32:733–4. doi: 10.1016/j.cub.2022.01.027 35134350

[164] Luo Y Song L Wang X Huang Y Liu Y Wang Q. Uncovering the mechanisms of cryptotanshinone as a therapeutic agent against hepatocellular carcinoma. Front Pharmacol (2020) 11:1264. doi: 10.3389/fphar.2020.01264 32903546 PMC7438559

[165] Shi D Zhao P Cui L Li H Sun L Niu J. Inhibition of PI3K/AKT molecular pathway mediated by membrane estrogen receptor GPER accounts for cryptotanshinone induced antiproliferative effect on breast cancer SKBR-3 cells. BMC Pharmacol Toxicol (2020) 21:32. doi: 10.1186/s40360-020-00410-9 32357920 PMC7193699

[166] Thorpe LM Yuzugullu H Zhao JJ. PI3K in cancer: divergent roles of isoforms, modes of activation and therapeutic targeting. Nat Rev Cancer (2015) 15:7–24. doi: 10.1038/nrc3860 25533673 PMC4384662

[167] Mendoza MC Er EE Blenis J. The ras-ERK and PI3K-mTOR pathways: cross-talk and compensation. Trends Biochem Sci (2011) 36:320–8. doi: 10.1016/j.tibs.2011.03.006 PMC3112285 21531565

[168] Li N Yang L Zhang B Chen S. Tanshinone IIA effects on ovarian cancer cell line. J Pharm Pharmacol (2018) 70:1369–77. doi: 10.1111/jphp.12961 29943422

[169] Zhang HS Zhang FJ Li H Liu Y Du GY Huang YH. Tanshinone II A inhibits human esophageal cancer cell growth through miR-122-mediated PKM2 down-regulation. Arch Biochem biophys (2016) 598:50–6. doi: 10.1016/j.abb.2016.03.031 27040384

[170] Ma J Li Y Wu M Zhang C Che Y Li W. Serum immune responses in common carp (Cyprinus carpio l.) to paraquat exposure: The traditional parameters and circulating microRNAs. Fish shellfish Immunol (2018) 76:133–42. doi: 10.1016/j.fsi.2018.02.046 29499338

[171] Huang M Lou D Cai Q Chang X Wang X Zhou Z. Characterization of paraquat-induced miRNA profiling response in hNPCs undergoing proliferation. Int J Mol Sci (2014) 15:18422–36. doi: 10.3390/ijms151018422 PMC4227223 25314302

[172] Kim HR Shin DY Chung KH. A review of current studies on cellular and molecular mechanisms underlying pulmonary fibrosis induced by chemicals. Environ Health Toxicol (2018) 33:e2018014–0. doi: 10.5620/eht.e2018014 PMC6182244 30286590

[173] Smith-Vikos T Slack FJ. MicroRNAs and their roles in aging. J Cell Sci (2012) 125:7–17. doi: 10.1242/jcs.099200 22294612 PMC3269020

[174] Wang G Dong F Xu Z Sharma S Hu X Chen D. MicroRNA profile in HBV-induced infection and hepatocellular carcinoma. BMC Cancer (2017) 17:805. doi: 10.1186/s12885-017-3816-1 29191172 PMC5709924

[175] Chen H Huang Y Huang J Lin L Wei G. Gigantol attenuates the proliferation of human liver cancer HepG2 cells through the PI3K/Akt/NF-κB signaling pathway. Oncol Rep (2017) 37:865–70. doi: 10.3892/or.2016.5299 27959444

[176] Juaid N Amin A Abdalla A Reese K Alamri Z Moulay M. Anti-hepatocellular carcinoma biomolecules: Molecular targets insights. Int J Mol Sci (2021) 22. doi: 10.3390/ijms221910774 PMC8509806 34639131

[177] Huang X Deng H Shen QK Quan ZS Tanshinone IIA. Pharmacology, total synthesis, and progress in structure-modifications. Curr Med Chem (2022) 29:1959–89. doi: 10.2174/0929867328666211108110025 34749607

[178] Zeng LW Zhou CX Liu JD Liu CH Mo JX Hou AF. Design, synthesis, and antimicrobial activities of new tanshinone IIA esters. Natural prod Res (2016) 30:2662–8. doi: 10.1080/14786419.2016.1138302 26829106

[179] Ren ZH Tong YH Xu W Ma J Chen Y. Tanshinone II a attenuates inflammatory responses of rats with myocardial infarction by reducing MCP-1 expression. Phytomedicine (2010) 17:212–8. doi: 10.1016/j.phymed.2009.08.010 19800776

[180] Lu XL Cai JT. Progress in research on anticancer effect of tanshinone II A and its mechanisms both *in vivo* and *in vitro* . Cancer Res Clin (2014) 26(12):854–6.

[181] Yao ZJ Guo R Zhang CS Liang PR. Study on the sodium tanshinone II a sulfonate and its synthesis technics. Natural Product Research and Development (2009) 21(03). doi: 10.16333/j.1001-6880.2009.03.007

[182] Pan ZG Zhang JT Duan LH Yang CI. Preparation of tanshinone II a derivative phenanthro [1,2-b]furan-10,11-methylene-dioxy-6,7,8,9-tetrahydro-1,6,6-trimethyl. Contemporary Chemical Industry (2014) 43(06). doi: 10.13840/j.cnki.cn21-1457/tq.2014.06.028

[183] Hagmann WK. The many roles for fluorine in medicinal chemistry. J med Chem (2008) 51:4359–69. doi: 10.1021/jm800219f 18570365

[184] Guo R Li L Su J Li S Duncan SE Liu Z. Pharmacological activity and mechanism of tanshinone IIA in related diseases. Drug design Dev Ther (2020) 14:4735–48. doi: 10.2147/DDDT.S266911 PMC7653026 33192051

[185] Wang T Wang C Wu Q Zheng K Chen J Lan Y. Evaluation of tanshinone IIA developmental toxicity in zebrafish embryos. Molecules (2017) 22. doi: 10.3390/molecules22040660 PMC6154573 28430131

[186] Li Z Zou J Cao D Ma X. Pharmacological basis of tanshinone and new insights into tanshinone as a multitarget natural product for multifaceted diseases. BioMed Pharmacother (2020) 130:110599. doi: 10.1016/j.biopha.2020.110599 33236719

[187] Ying Q Teng Y Zhang J Cai Z Xue Z. Therapeutic effect of tanshinone IIA on liver fibrosis and the possible mechanism: A preclinical meta-analysis. Evidence-Based complement Altern med.: eCAM (2019) 2019:7514046. doi: 10.1155/2019/7514046 PMC6930756 31915451

[188] Petroni G Formenti SC Chen-Kiang S Galluzzi L. Immunomodulation by anticancer cell cycle inhibitors. Nat Rev Immunol (2020) 20:669–79. doi: 10.1038/s41577-020-0300-y PMC7584736 32346095

[189] Zhou ZY Zhao WR Zhang J Chen XL Tang JY. Sodium tanshinone IIA sulfonate: A review of pharmacological activity and pharmacokinetics. BioMed Pharmacother (2019) 118:109362. doi: 10.1016/j.biopha.2019.109362 31545252

[190] Wang Y Feng NP Nan YL Zhong LH Chen HY Fan ZZ. [Study on the pharmacokinetics of tashinone II(A) -loaded polylactic acid nanoparticles in rabbit]. Zhong yao cai = Zhongyaocai = J Chin med mater (2011) 34:1392–5. 22260008

